# Modeling early germline immunization after horizontal transfer of transposable elements reveals internal piRNA cluster heterogeneity

**DOI:** 10.1186/s12915-023-01616-z

**Published:** 2023-05-24

**Authors:** Amna Asif-Laidin, Karine Casier, Zoheir Ziriat, Antoine Boivin, Elise Viodé, Valérie Delmarre, Stéphane Ronsseray, Clément Carré, Laure Teysset

**Affiliations:** 1 Sorbonne Université, CNRS, Institut de Biologie Paris-Seine, Laboratoire Biologie du Développement, UMR7622, “Transgenerational Epigenetics & Small RNA Biology”, Paris, F-75005 France; 2 Present Address: CNRS, Institut de Biologie Physico-Chimique, Laboratoire de Biologie Moléculaire et Cellulaire des Eucaryotes, UMR8226, Telomere Biology, Paris, F-75005 France

**Keywords:** Transposable elements, piRNA clusters, PIWI-interacting RNA, Subtelomeres, *Drosophila*, Horizontal transfer, Transgenerational inheritance

## Abstract

**Background:**

A fraction of all genomes is composed of transposable elements (TEs) whose mobility needs to be carefully controlled. In gonads, TE activity is repressed by PIWI-interacting RNAs (piRNAs), a class of small RNAs synthesized by heterochromatic loci enriched in TE fragments, called piRNA clusters. Maintenance of active piRNA clusters across generations is secured by maternal piRNA inheritance providing the memory for TE repression. On rare occasions, genomes encounter horizontal transfer (HT) of new TEs with no piRNA targeting them, threatening the host genome integrity. Naïve genomes can eventually start to produce new piRNAs against these genomic invaders, but the timing of their emergence remains elusive.

**Results:**

Using a set of TE-derived transgenes inserted in different germline piRNA clusters and functional assays, we have modeled a TE HT in *Drosophila melanogaster*. We have found that the complete co-option of these transgenes by a germline piRNA cluster can occur within four generations associated with the production of new piRNAs all along the transgenes and the germline silencing of piRNA sensors. Synthesis of new transgenic TE piRNAs is linked to piRNA cluster transcription dependent on Moonshiner and heterochromatin mark deposition that propagates more efficiently on short sequences. Moreover, we found that sequences located within piRNA clusters can have different piRNA profiles and can influence transcript accumulation of nearby sequences.

**Conclusions:**

Our study reveals that genetic and epigenetic properties, such as transcription, piRNA profiles, heterochromatin, and conversion efficiency along piRNA clusters, could be heterogeneous depending on the sequences that compose them. These findings suggest that the capacity of transcriptional signal erasure induced by the chromatin complex specific of the piRNA cluster can be incomplete through the piRNA cluster loci. Finally, these results have revealed an unexpected level of complexity that highlights a new magnitude of piRNA cluster plasticity fundamental for the maintenance of genome integrity.

**Supplementary Information:**

The online version contains supplementary material available at 10.1186/s12915-023-01616-z.

## Background

Transposable elements (TEs) are mobile endogenous genetic elements predominantly transmitted through vertical transfer (i.e., from one generation to the next) like any other gene. In most metazoan germlines, transposition is repressed during gametogenesis by specialized 23–29-nt small RNAs associated with PIWI proteins [1–3]. These small RNAs have been named PIWI-interacting RNAs or piRNAs. They are produced from numerous heterochromatic loci, called piRNA clusters, that mainly contain TE fragments coming from ancient insertions serving as libraries of mobile sequences to repress. Among the 140 ovarian piRNA clusters of *Drosophila melanogaster*, only a few of them (the *flamenco* somatic and the *42AB*, *38C*, *80F*, *20A* germline clusters) have been extensively studied to identify factors involved in piRNA-dependent silencing [4–9]. It is well established that germline piRNA clusters are transcribed by a specialized RNA polymerase II complex that contains Moonshiner (Moon) recruited to the locus through its interaction with a complex containing the HP1 homolog Rhino (Rhi), Deadlock (Del), and Cutoff (Cuff) forming the RDC complex [6, 10]. Furthermore, only maternally inherited piRNAs participate in the transgenerational memory of TE sequences to repress as no paternally inherited piRNAs have been found [11, 12]. Although our understanding of the molecular events involved in the maintenance of active piRNA clusters through generations has expanded substantially, major gaps still exist especially in the early events of functional piRNA cluster establishment.

In gonads, 21-nt siRNAs can be also synthesized from dual-stranded piRNA clusters [13]. They are usually produced from double-stranded transcripts that are recognized by the Dcr-2 nuclease, and once loaded on the Ago2 protein, they can induce the cleavage of complementary RNA targets [14]. However, their germline function is not clear as they can be dispensable without significantly affecting viability, fertility, TE repression, and piRNA cluster maintenance [15].

Rarely, TEs are also transmitted through horizontal transfer (HT) corresponding to DNA transmission between unrelated individuals. It has been however noted that HTs are more frequent than originally thought [16–18] raising the question of how and by which dynamics new piRNAs are produced by naïve genomes in the absence of maternal inheritance of complementary piRNAs. One of the best documented HT is the one of *P* element that has successfully invaded the genome of natural populations of *D. melanogaster* within two decades during the twentieth century and of *D. simulans* since the beginning of the twenty-first century [19–21]. In *D. melanogaster*, the subtelomere of the *X* chromosome (cytological site *1A*) is a hot spot of *P* insertions [22–25]. This locus also known as *telomeric-associated sequences* (*X-TAS*) is also one of the *Drosophila* piRNA clusters (hereafter called *cluster 1A*) that can be dispensable in laboratory environments [26]. *Cluster 1A* contains repeats with regions sharing homologies with the autosomal subtelomeric piRNA clusters of the *2R* and the *3R* chromosomes (*clusters 60F* and *100F*) and a 0.9-kb region called *T3* not present elsewhere in the genome, a unique feature among all known piRNA clusters [26]. From a *P* copy inserted in *cluster 1A*, piRNAs derived from *P* are produced in the female germline capable of repressing euchromatic active *P* elements [11, 27–31]. Moreover, *lacZ* encoding *P* transgenes inserted in *cluster 1A* (e.g., *P(lArB)* in *P-1152* strain) have been shown to silence female germline expression of another *P-lacZ* transgene located in euchromatin [32, 33]. This euchromatic *P-lacZ* has served as a reliable reporter system (or “piRNA sensor”) for studying functional piRNA biology as its silencing depends on piRNA biogenesis factors [5, 12, 34–38] but not on siRNA biogenesis factors [15, 34]. Indeed, using this sensor, we have shown that the mechanism of repression is accomplished according to an ON/OFF mode, where egg chambers show either strong (ON) or no (OFF) *lacZ* silencing [34]. We have also shown that when subtelomeric *P(lArB)* transgenes were paternally inherited, the number of fully repressed egg chambers in the first generation is low and increases progressively to reach a maximum level of repression after five generations [34].

We have also found that a naïve locus made of seven tandemly repeated *P(lacW)* transgenes, in the strain *BX2*, that is maintained as a non-piRNA producer over the years, can be fully converted into a stable piRNA cluster in one generation by maternally inherited piRNAs matching the whole length of the transgenes, uncovering a stable case of epigenetic conversion called paramutation [12]. The switch from a naïve locus to an active one able to produce piRNAs, hereafter referred to as “conversion,” is associated with an enrichment of H3K9me3 [5, 39]. Such functional conversion can occur when the locus producing maternal piRNAs is located on different chromosomes and is partially homologous to *P(lacW)* [15]. Moreover, ovarian small RNA analyses revealed that conversion of the full length of *P(lacW)* can be completed when tested after the third generation [15]. These results along with others suggested that the piRNA machinery is able to eventually co-opt an unknown sequence from the maternal piRNA repertoire to produce de novo piRNAs of this new sequence [15, 40–42]. A similar scenario could happen when a naïve genome faces HT of new TEs that insert into piRNA clusters. At first, the TE copy newly integrated into a piRNA cluster is surrounded by sequences that are targeted by maternal piRNAs (Additional file 1: Fig. S1). In fine, new piRNAs against this TE will be synthesized and able to repress active euchromatic copies. The rareness of such event and the repetitive nature of piRNA clusters have made it difficult to directly address the precise latency and the identification of early molecular events involved in these co-option processes (Additional file 1: Fig. S1C).

We report here a study where we have modeled a TE *neo* insertion into a piRNA cluster in a naïve genome to question the kinetics of production of new specific piRNAs and their capacity to repress from the first generation, necessary to protect genome integrity. To model such event, we have used several transgenes, derived from the *P* transposon, inserted in different piRNA clusters or inserted in euchromatin working as piRNA sensors. Using the paternal origin of transgenes inserted in piRNA clusters, we have been able to correlate the emergence of new piRNA production with their silencing capacities using functional assays from the very first generation. We have also shown that the kinetics of co-option by piRNA cluster leading to the conversion of a sequence could depend on intrinsic properties such as its length. We have identified that all regions of the transgene are converted concomitantly with the same efficiency at each generation, but this conversion is restricted to sequences nested in piRNA clusters as previously shown [43] revealing an active mechanism preventing cis-propagation of piRNA clusters to their flanking regions [44]. By studying more specifically a germline subtelomeric piRNA cluster, *cluster 1A*, from the *P-1152* strain containing *P(lArB)* and *T3* sequences, we have identified that heterogeneity can be observed inside piRNA clusters as they can exhibit different rates of conversion and different piRNA profiles (symmetrical and asymmetrical dual-strand clusters) associated with chromatin and transcription variations along the locus. Altogether, this study brings new insights into piRNA cluster dynamics.

## Results

### Functional conversion of paternally inherited subtelomeric transgenes completed within four generations is associated with piRNA synthesis

It was previously observed that silencing of a *lacZ* sensor induced by subtelomeric *P(lArB)* transgenes inserted in *cluster 1A* was female germline-specific, with a maternal effect that showed variegated ON/OFF *lacZ* egg chambers repression (between 80 and 100% of repressed egg chambers) and dependent on the piRNA biogenesis pathway [12, 34, 36, 38, 45]. By contrast, paternally inherited *P(lArB)* induced *lacZ* silencing in few ovarian egg chambers in the first generation (between 5 and 35%) that increased in subsequent generations [34]. The progressive increase in the number of repressed egg chambers per ovary suggested that the amount of *lacZ* piRNAs per ovary produced by the subtelomeric *P(lArB)* was proportionally increasing at each generation.

**Fig. 1:**
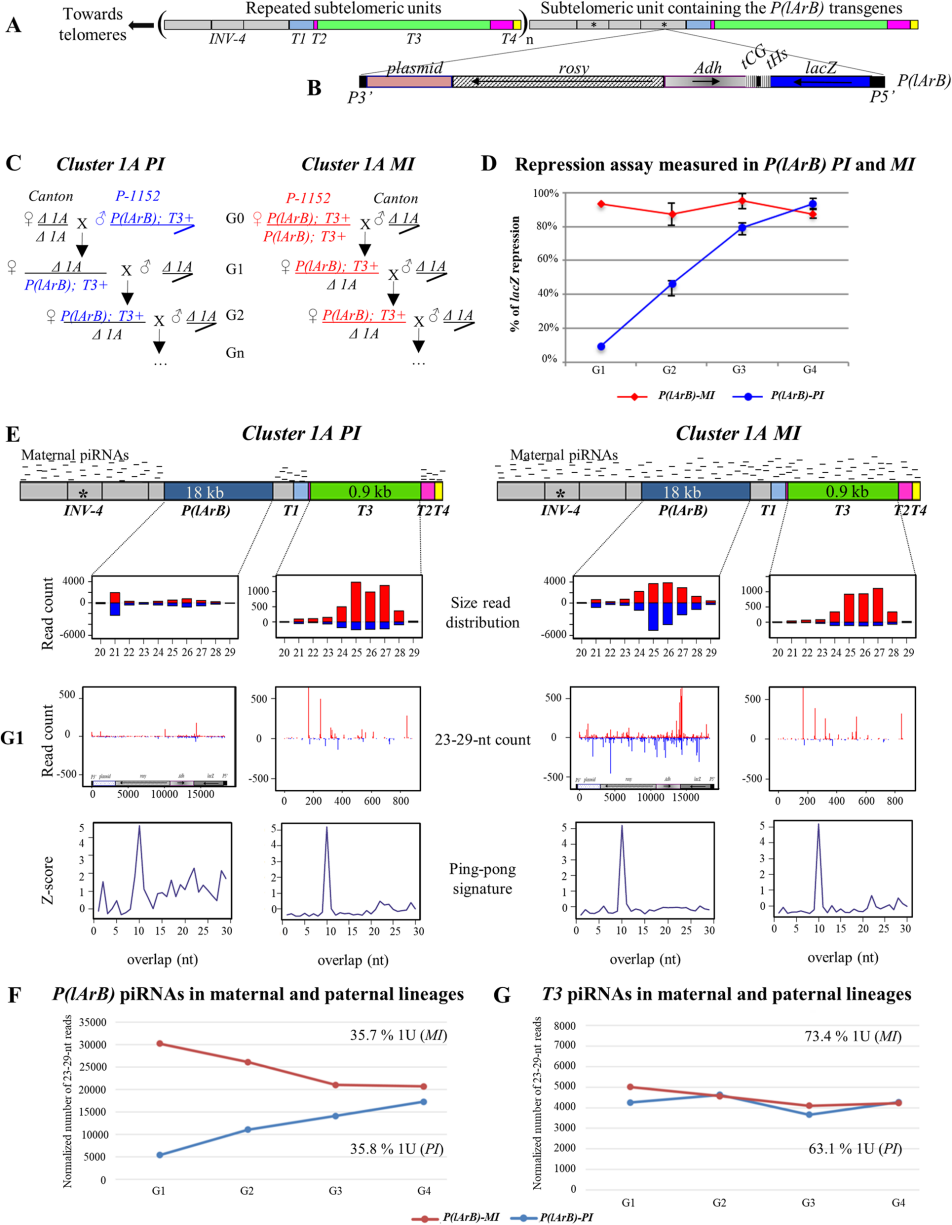
Heterogeneity inside *cluster 1A*. **A** Subtelomeric *cluster 1A* on the *X* chromosome in the *P-1152* strain composed of several repeats (*n*) containing solo LTRs of *INV-4* (gray), *T1* (blue), *T2* (pink), and *T4* (yellow) domains also found in autosomal subtelomeric piRNA clusters and the *T3* domain (0.9 kb) found only in *cluster 1A* (green). One of the repeats contains the *P(lArB)* transgenes (18 kb) (asterisks). **B** The *P(lArB)* transgene includes the 5′ and 3′ *P*-derived sequences, a plasmid sequence, *rosy*, *Adh*, *lacZ* (under the control of the *P* promoter), and the two transcriptional terminators (tCG and tHs). Black arrows represent the sense of transcription. **C** Paternal (*PI*) and maternal (*MI*) inheritances of *cluster 1A* were obtained by two reciprocal crosses between *P-1152* and *Canton* strains. The maternal alleles are above the fraction. *P-1152* carries *P(lArB)* and *T3* in *cluster 1A* (“*P(larB); T3* + ”), absent in *Canton* (“*Δ-1A*”). **D** Ovarian *lacZ* repression in the *PI* and *MI P(lArB)* lineages. Values represent the mean of four sublines with standard deviation. **E** Experimental design showing regions complementary to maternal piRNAs (small black lines) in G1. Maternal piRNAs in *PI* are produced by autosomal subtelomeres. Below are the size distributions of normalized 20–29-nt counts and the relative frequency (*z*-score) of overlapping sense-antisense small RNA pairs in the subsets of 23–29-nt RNAs matching *P(lArB)* and *T3*, showing enrichment of 10 nucleotides overlaps. The sense piRNAs are in red, and the antisense are in blue. **F**, **G** Normalized 23–29-nt reads mapping to *P(lArB)* (**F**) and *T3* (**G**) of the *MI* H and *PI* D sublines. The percentage of 23–29-nt *P(lArB)* and *T3* RNAs beginning with a 5′ uridine (1U bias) and characteristic of piRNAs are indicated for G4. Note that *P(lArB)* (18 kb), *X* subtelomeric repeat (1.8 kb), and *T3* (0.9 kb) are not drawn to scale

**Fig. 2:**
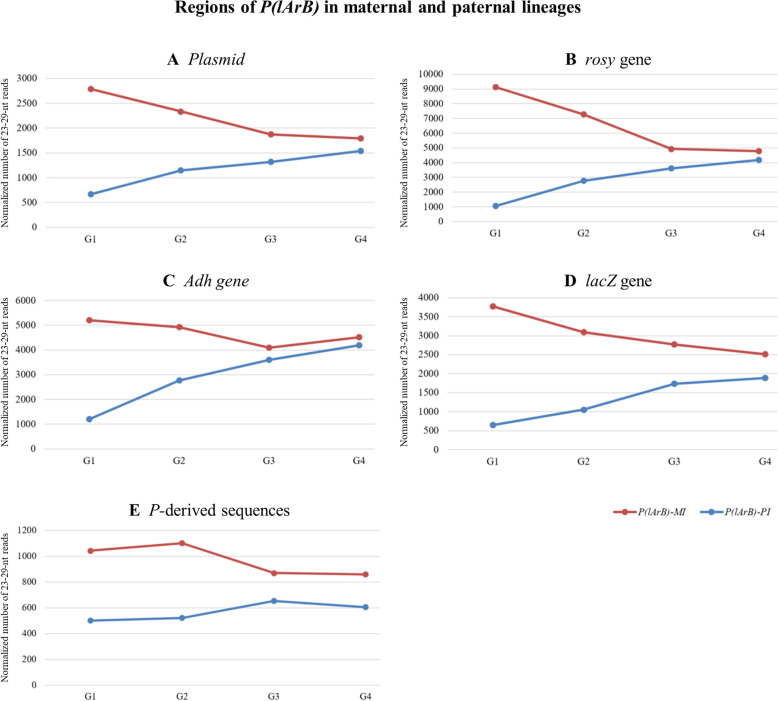
Normalized 23–29-nt reads on *P(lArB)* regions in *MI* and *PI* lineages. Normalized ovarian 23–29-nt reads mapping to different regions of *P(lArB)*: *plasmid* (**A**), *rosy* (**B**), *Adh* (**C**), *lacZ* (**D**) genes, and the *P*-derived sequences (**E**) in the *MI* H and *PI* D sublines (Additional file 2: Table S3). All the sequences, either exogenous (*lacZ*, *plasmid*, and *P*) or endogenous (*rosy*, *Adh*) from the *Drosophila* genome, have similar kinetics of producing a progressive increase number of 23–29-nt small RNAs over generations

To test this model, we have set up reciprocal crosses between the *P-1152* and the *Canton* strains. *P-1152* contains two *P(lArB)* transgenes inserted in *cluster 1A* (Fig. 1A, B). *Canton* lacks *cluster 1A* (*Δ-1A* strain) and is devoid of *P*-derived transgenes (Additional file 1: Fig. S2A, Table 1, Additional file 2: Table S1 [26]). However, *Canton* is carrying the autosomic subtelomeric piRNA clusters (*clusters 60F* and *100F*) that produce piRNAs targeting the common regions between *cluster 1A* and the autosomal clusters (*T1*, *T2*, *T4*, and *INV-4*, Fig. 1A [26]). For these crosses, *P(lArB)* were first paternally or maternally inherited and then maternally maintained as hemizygous in the successive generations to establish the paternal and the maternal lineages (*PI* and *MI*, respectively) (Fig. 1C). Four independent replicate lines were generated for each lineage. Ovarian *lacZ* silencing was assayed at each generation by crossing *PI* and *MI* females with males containing the *lacZ* sensor (Additional file 1: Fig. S2B). Ovarian X-gal staining of the progenies allowed to quantify the level of conversion of *P(lArB)* into an active piRNA-producing locus. When *P(lArB)* were paternally inherited (*PI*), the first generation showed a limited amount of repressed egg chambers (G1; 9.25%) that progressively reached the same level of repression as the maternal lineage after four generations (> 90%, Fig. 1D and Additional file 2: Table S2), reminiscent of previous results with another *lacZ* sensor [34]. In parallel, to correlate the level of repression with the production of subtelomeric *P(lArB)* piRNAs, ovarian small RNAs were sequenced, and the normalized 23–29-nt *P(lArB)* small RNAs were analyzed at each generation. At G1, the number of 23–29-nt small RNAs in *PI* females was low compared to *MI* females increasing progressively at each generation reaching a plateau at G4 corresponding to the same amount of 23–29-nt small RNAs than in *MI* females (Fig. 1E, F, Additional file 1: Fig. S3A and Additional file 2: Table S3). This correlates with the *lacZ* repression (Fig. 1D). The enrichment in uridine as the first nucleotide for the 23–29-nt small RNAs (1U bias) (Fig. 1F) and an enrichment of ping-pong pairs (Fig. 1E), two signatures of germline piRNA biogenesis, together with previous mutant analyses reinforced the assumption that these small RNAs are genuine piRNAs [5, 12, 34, 36, 38]. Therefore, these results correlate the *lacZ* silencing efficiency with piRNA amount. Furthermore, the distribution of piRNAs on sense and antisense along *P(lArB)* corresponds to a dual-strand piRNA cluster profile (Fig. 1E). These results were reproduced in different genetic backgrounds (*Canton* and *w*^*1118*^, Additional file 1: Fig. S4) and with another *P* derived transgene inserted in the autosomal subtelomeric piRNA cluster *100F* (Additional file 1: Fig. S5). Interestingly, piRNAs synthesis was homogeneously increasing for all regions whatever their position within *P(lArB)* in *PI*, with roughly the same kinetics over generations. The most distal parts of the transgene, adjacent to sequences targeted by maternally inherited piRNAs, do not show a quicker conversion process than the internal parts. These results support the hypothesis that the kinetics of conversion was roughly the same independently of their position within the transgene, their sequence origin (*Drosophila* or *E. coli*), or their nature (genes or *P*-derived sequences) (Fig. 2, Additional file 1: Fig. S3C-G). This is in accordance with the capacity of Rhino and its partners to erase internal transcriptional signals, leading most likely to a uniform production of piRNAs independently of the sequence origin [4, 6, 46]. Taken together, these results support the idea that the percentage of egg chambers in an ON or OFF state for *lacZ* expression (Fig. 1D) reflects the piRNA amount across paternal inheritance (Fig. 1F).

**Table 1:** Strains used in this study

**Strain**	**Transgene of interest**	**Region of interest in the sequence**	**Localization**
*P-1152*	*P(lArB*), a *P*-derived transgene	*P:* The 5′ and 3′ regions of the *P* element	Inserted in c*luster 1A*
		*l*: *lacZ* gene	
		*A*: Adh gene	
		*r*: *rosy* gene	
		*B*: plasmid	
		*T3*	In *cluster 1A*
*BX2*	*P(lacW)*, a *P*-derived transgene	*P*: The 5′ and 3′ regions of the *P* element	Inserted in the left arm of the second chromosome (*50C*)
		*lac*: *lacZ* gene	
		*W*: white gene	
		Plasmid sequence	
*RS3*	*P(RS3)*, a *P*-derived transgene	*P*: The 5′ and 3′ regions of the *P* element	Inserted in *cluster 100F*, the *3R* subtelomere
		*FRT*: sequence of flippase recognition target (not used in this study)	
		*white* gene	
*Oregon*	No transgene	*T3*	*In cluster 1A*
*Canton*	No transgene		No* cluster 1A*
*w*^*1118*^	No transgene		No* cluster 1A*

Consistent with an HT of TE into a naïve genome, G1 males of *P(lArB)-PI* contribute only to DNA transgenic copies without contributing to complementary piRNAs inheritance. Moreover, our results suggest that maternally inherited piRNAs produced by the autosomal subtelomeres in *Δ-1A* females can target and convert progressively across generations the subtelomeric repeats surrounding *P(lArB)* insertions (Fig. 1E, F and Additional file 1: Fig. S1).

### *P(lArB)* and *T3* show distinct conversion dynamics

*Cluster 1A* is composed of repeats shared with other piRNA clusters in autosomal subtelomeres and of a unique 0.9-kb *T3* domain not found outside of *cluster 1A* (Fig. 1A) [26]. In view of the progressively increasing number of *P(lArB)* piRNAs in the paternal lineage (Fig. 1F), we hypothesized the same dynamic over four generations for a paternally inherited *T3* domain. To test this, ovarian small RNA libraries were reanalyzed by aligning the 23–29-nt reads to the *T3* domain. Unexpectedly, the same amount of normalized *T3* 23–29-nt small RNAs was found in both paternal and maternal lineages from the first generation (Fig. 1E, G, Additional file 1: Fig. S3B and Additional file 2: Table S3). This was observed using the *Canton* or *w*^*1118*^* Δ-1A* strain genetic background (Additional file 1: Fig. S4D). As in our previous work, the 23–29-nt *T3* small RNAs were mostly produced from one strand leading to an asymmetrical dual-strand piRNA cluster, showing an enrichment in uridine at first nucleotide (1U bias) [26] and an overlapping of 10 nucleotides bias among the small RNA pairs (a ping-pong signature, Fig. 1E, G).

**Fig. 3:**
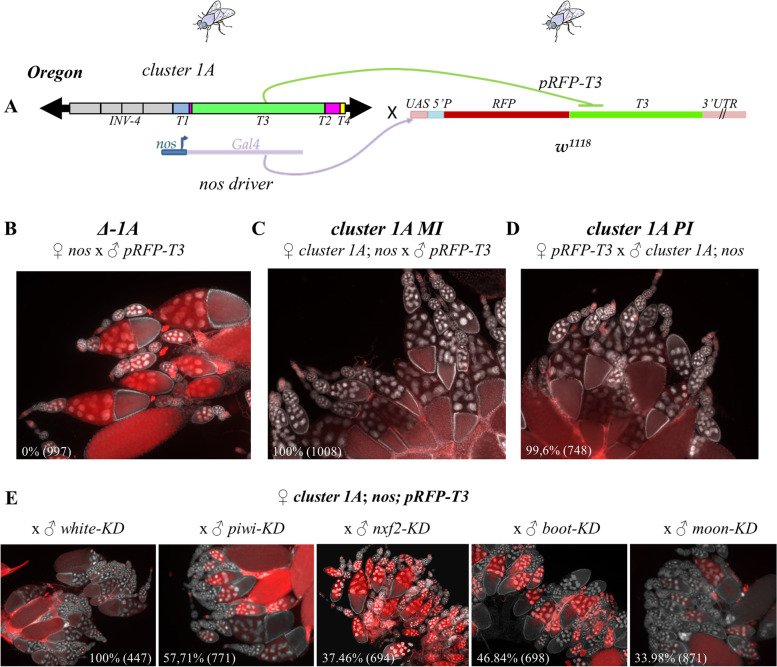
*Cluster 1A* relies on the germline piRNA pathway. **A** Schematic representation of the experimental cross: *Oregon* flies containing *cluster 1A* carrying *T3* and the *nos-Gal4* germline driver were crossed with *w*^*1118*^ flies devoid of *cluster 1A* (*Δ-1A*) but expressing the *pRFP-T3* sensor. **B** Strong ovarian germline *RFP* expression of progenies from control *nos-Gal4* females crossed with males encoding the *pRFP-T3* sensor in the absence of *cluster 1A*. Maternally (**C**) or paternally (**D**) inherited *T3* strongly represses ovarian germline expression of the *pRFP-T3* piRNA sensor. **E** Ovarian *pRFP-T3* repression of maternally inherited *T3* is strongly affected by germline knockdown of *piwi*, *nxf2*, *boot*, and *moon* (*piwi-KD*, *nxf2-KD*, *boot-KD*, *moon-KD*). Knockdown for *white* served as control. Repression was assayed by counting the percentage of RFP-silenced egg chambers at stages 8–10. The total numbers of counted egg chambers are indicated in parenthesis. *RFP* expression is in red, and *DAPI* staining, indicating nuclei, is in white. Parental crosses are indicated above micrographs

To test if *T3* small RNAs were functional for repression from the first generation, we designed a *P*-derived transgenic sensor using the red fluorescent protein (*RFP*) reporter gene transcriptionally fused to the *T3* domain and expressed under the control of the *UASp* sequences (*pRFP-T3*). *RFP* expression was induced in female germline by the Gal4 protein expressed under the control of the *nanos* (*nos*) promoter (Fig. 3A). Female germline expression of *pRFP-T3* was observed in the *Δ-1A* *w*^*1118*^ background confirming the absence of other *T3* piRNA sources (Fig. 3B). When the *P-1152* strain was used as a donor of *T3* small RNAs, almost complete silencing of *pRFP-T3* was observed induced by small RNAs produced by both the *T3* domain and *P-*derived sequences of *P(lArB)* (Additional file 1: Fig. S6A and B). To avoid this, we used the *Oregon* strain, as a donor of *T3* small RNAs [26], because this strain is devoid of *P*-derived sequences (*P* transgene or natural* P* element) and carries *cluster 1A* [1, 26]. Ovarian *pRFP-T3* expression was repressed when *cluster 1A* of the *Oregon* strain was maternally inherited (100% of RFP repressed egg chambers (*n* = 1008), Fig. 3C and Additional file 1: Fig. S6C) suggesting that small RNAs produced from *T3* are indeed fully functional germline repressors. Moreover, the paternally inherited *T3* locus from *Oregon* was also able to strongly repress *pRFP-T3* from the first generation (99.6% of RFP repressed egg chambers (*n* = 748), Fig. 3D). Unlike *P(lArB)*, *T3* paternal allele is functionally converted in a single generation in all cells, and in direct correlation with the amount of *T3* small RNAs detected in *PI* G1 females (Fig. 1E, G and Additional file 1: Fig. S3B). To confirm that the *pRFP-T3* silencing was piRNA mediated, we knocked down germline expression of *Piwi* and *Nxf2*, two co-transcriptional silencing factors of the piRNA pathway, *Bootlegger* (*Boot*) that recruits nuclear export factors like Nxf3-Nxt1 to piRNA cluster loci [1, 47] and *Moon*, a subunit specific of germline piRNA cluster RNA polymerase [10]. Figure 3E shows that *RFP* silencing was strongly affected by the knockdown of these factors supporting the notion that 23–29-nt *T3* small RNAs are functional piRNAs targeting *pRFP-T3* reporter in the female germline. These knockdowns were also affecting *lacZ* sensor silencing induced by subtelomeric *P(lArB)* (Additional file 1: Fig. S7).

Thus, paternally inherited *P(lArB)* and *T3* are piRNA-producing sequences that display different rates of conversion as well as different piRNA distribution profiles (symmetric dual-strand for *P(lArB)* and asymmetrical dual-strand for *T3*), although they are located in the same locus and dependent on the same piRNA pathway. Functionally, these results might indicate that piRNA cluster activation is dependent on maternal piRNA inheritance at each generation that targets the flanking subtelomeric regions (Additional file 1: Fig. S1), but also on properties of sequences present within the locus.

### Sequence length could influence the conversion efficiency

**Fig. 4:**
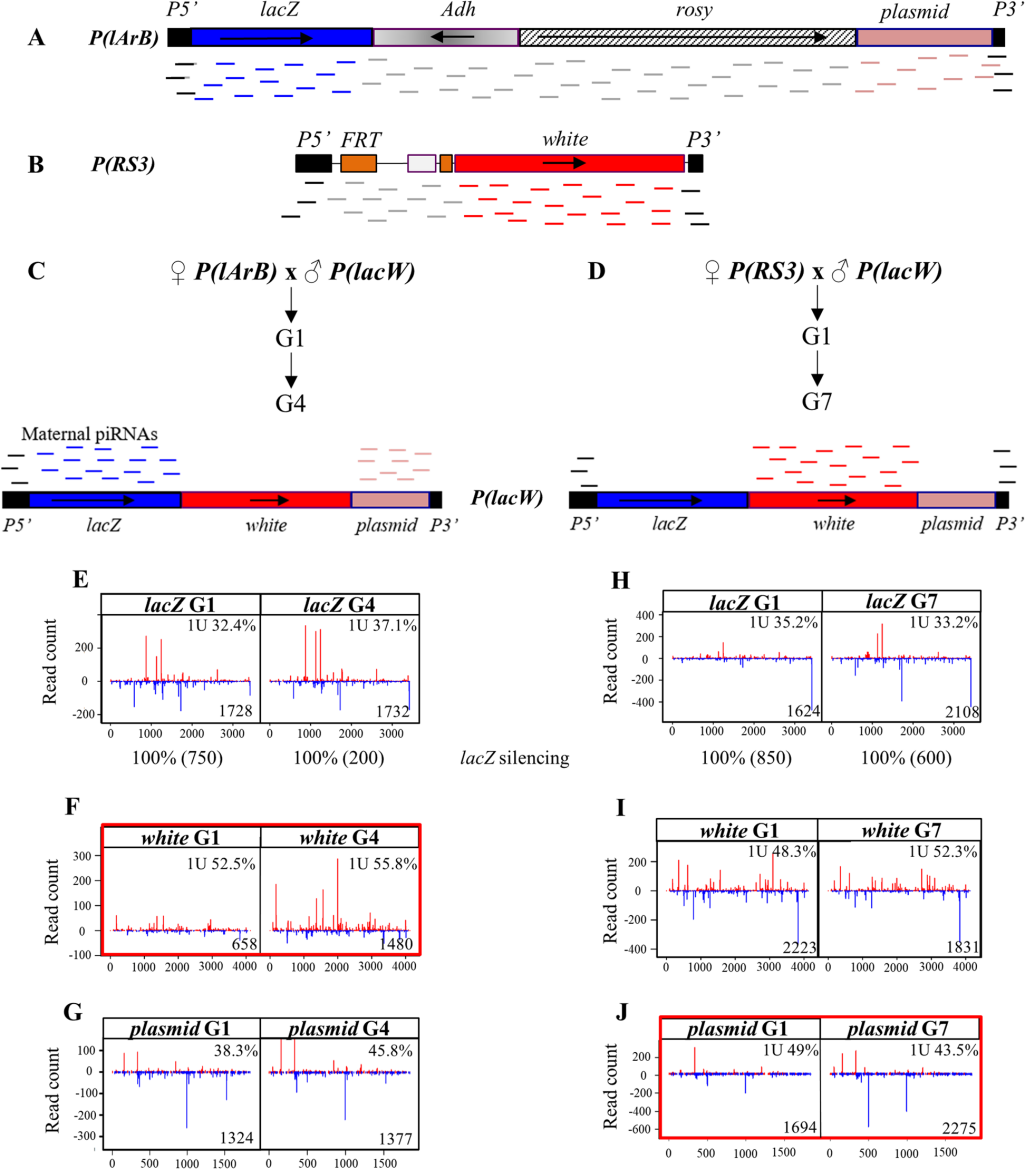
Conversion of the *P(lacW)* transgenes by *P(lArB)* or *P(RS3)*. Diagrams of *P(lArB*) inserted in *cluster 1A* (**A**) and *P(RS3)* inserted in *cluster 100F* (**B**). piRNAs produced by both clusters are represented by small colored lines below the transgenes. Crosses to convert the seven *P(lacW)* transgenes inserted in tandem by *P(lArB)* (**C**) or by *P(RS3)* (**D**). Complementary maternal piRNAs produced by either *P(lArB)* or *P(RS3)* are indicated above the *P(lacW)* scheme. Normalized reads of 23–29-nt mapping to *lacZ* (**E**, **H**), *white* (**F**, **I**), and plasmid sequence (**G**, **J**). When *P(lacW)* transgenes are activated by *P(lArB)*, the density of 23–29-nt small RNAs between G1 and G4 is similar for *lacZ* (**E**) and the plasmid sequence (**G**) and 2.25-fold higher for *white* (red box, **F**). When *P(lacW)* transgenes are activated by *P(RS3)*, the density ratio of 23–29-nt small RNAs is close to 1 between G1 and G7 for all the domains of *P(lacW)* (1.3 for *lacZ* (**H**), 0.8 for *white* (**I**) and 1.3 for the 1.8-kb plasmid region (red box, **J**) that is not targeted by maternal piRNAs in G1). The density of normalized 23–29-nt reads per kb (reads/kb) and the fraction of 1U bias at 5′ are indicated in each panel. The *P(lArB)* (18 kb), *P(RS3)* (6 kb), and *P(lacW)* (10.7 kb) transgenes are not drawn to scale

The contrasting conversion rate observed in G1 between the paternally inherited 0.9-kb *T3* domain and the 18-kb *P(lArB)* suggests that short sequences could be converted more efficiently than longer ones. To test whether the sequence length could influence the frequency of conversion, we used several strains: the seven tandemly repeated *P(lacW)* transgenes in the *BX2* strain that can be converted into an active piRNA cluster by complementary maternal piRNAs [12, 15], the *P-1152* strain, and the *RS3* strain that carries the *P(RS3)* transgene inserted in the autosomal *3R* subtelomeric piRNA cluster, *cluster 100F* (Fig. 4, Table 1 and Additional file 2: Table S1). Both the *P(lArB)* and *P(lacW)* transgenes encode the 3.5-kb *E. coli lacZ* gene and the 1.8-kb bacterial plasmid backbone (Fig. 4A, C). The *P(RS3)* and *P(lacW)* transgenes both encode the 4.1-kb *white* gene (Fig. 4B, D). In addition, *P(lArB)*, *P(RS3)*, and *P(lacW)* have in common the 5′ (0.58 kb) and 3′ (0.23 kb) distal regions of the *P* element.

In this set of experiments, hemizygous *P(lArB)* and *P(RS3)* females (donors of piRNAs) were crossed to *BX2* males hemizygous for *P(lacW)* (Fig. 4 and Additional file 1: Fig. S8). The G1 progenies that paternally inherited the *P(lacW)* transgenes and maternally inherited the piRNAs from either *P(lArB)* or *P(RS3)*, but not the subtelomeric transgenes, were then crossed to each other for several generations (Fig. 4C, D and Additional file 1: Fig. S8). Previously, ovarian 23–29-nt RNAs from G3 up to G10 mapping to the different regions of *P(lacW)* were identified at a time when the complete conversion was reached, that is to say when stable paramutation already occurred [15]. Here, to question the conversion establishment, ovarian 23–29-nt RNAs were analyzed as soon as G1 and up to G4 or G7 generations. piRNAs matching *lacZ* and plasmid sequences maternally inherited from *P(lArB)* were able to convert in G1 the complementary regions in *P(lacW)* (Fig. 4E, G). The *lacZ* gene, expressed as a transcriptional fusion with *P* first exons, and the *white* gene of *P(lacW)* were also converted from the first generation by complementary piRNAs synthesized by the maternal *P(RS3)* allele (density ratio of 1.3 and 0.8 between G1 and G7, respectively, Fig. 4H, I, 100% of *lacZ* sensor silencing Fig. 4H and Additional file 1: Fig. S8). Strikingly, the piRNA population in the G1 progeny was limited for the 4.1 kb *white* sequence absent in *P(lArB)* (658 reads/kb) that significantly increased in G4 (1480 reads/kb) (ratio of 2.25 between G1 and G4, Fig. 4F), while the piRNAs for the 1.8-kb plasmid sequence not included in *P(RS3)* were already detected at G1 (ratio of 1.3 between G1 and G7, Fig. 4J).

Therefore, combining these results with the fact that the 18-kb *P(lArB)* transgene requires several generations to be fully converted when it is paternally inherited (Fig. 1) and that the 0.9-kb *T3* conversion occurs into only one generation, we suggest that the efficiency of conversion of a new sequence inserted into a piRNA cluster targeted by maternally inherited piRNAs, but not targeted itself, could depend on its length.

We have also tested whether the conversion rate could be influenced by the nucleotide composition of sequences. One hypothesis is that the *T3* and plasmid sequences might be enriched in some dinucleotides compared to the *P(lArB)* and *white* sequence. To address this, we have computed the dinucleotide content of the four sequences. We have found that the content of dinucleotides is quite consistent between sequences with different lengths. We only noticed a slight bias toward AT/TA dinucleotides for the *T3* sequence, but not for the others including the plasmid, that is converted with the same efficiency as *T3* (Additional file 1: Fig. S9A, Additional file 2: Table S5). To understand the potential role of this bias on sequence conversion rate, we have included it in a linear regression model (piRNA.density ~ sequence.length + AT or TA) but obtained poor *p*-values (0.665 and 0.199, respectively) to conclude. Therefore, it is unlikely that dinucleotide content, in itself, might explain the difference of conversion efficiency between the considered sequences.

Finally, we have tested if small RNAs with imperfect mapping (i.e., 3 mismatches) with the references sequences could exist in the parental strains that could participate to the one-generation conversion of *T3* and the plasmid, while absent or less abundant for *P(lArB)* and *white* gene. The parental strains are *Canton* and *w*^*1118*^ for *T3* and *P(lArB)* (Fig. 1 and Additional file 1: Fig. S4), *P-1152* for *white* gene included in *P(lacW)* transgene (Fig. 4F), and *RS3* for *plasmid* included in *P(lacW)* (Fig. 4J). For *T3*, 3 mismatch piRNAs were identified in the *Canton* and *w*^*1118*^ parental strains (1563 and 1378 reads/kb, respectively) (Additional file 1: Fig. S9B and C, Additional file 2: Table S6). This result was expected, as the *L* subtelomeric repeats (left arms of the autosomal chromosomes shown to be piRNA clusters [1, 26]) contain a small domain with similarities with *T3* [26]. It has been shown that the Piwi protein from a sponge specie can be tolerant to mismatches for the piRNA target binding but requires extensive pairing for the endonuclease activity preventing unwanted mRNA targeting [48]. In *Drosophila*, the 3 mismatches piRNAs mapping to *T3* in *Canton* and *w*^*1118*^ do not have a ping-pong signature (Additional file 1: Fig. S9F and G) and are not functional for the silencing of the *pRFP-T3* piRNA sensor (Fig. 3B, Additional file 1: Fig. S6B). It can be also noted that the region sharing similarities with the *L* subtelomeres does not show a high piRNA density of 0 mismatch *T3* piRNAs (see the read counts around position 600 in Fig. 1E, Additional file 1: Fig. S4D, S9B, C). This suggests that the *L* piRNAs do not participate to the conversion of the *T3* domain. Few 3 mismatch piRNAs were identified mapping to the plasmid in *RS3* (8.8 reads/kb), and with the same order of magnitude for the *white* gene in *P-1152* (5.8 reads/kb) and to *P(lArB)* in the *Canton*, *w*^*1118*^ (8.1 and 9.5 reads/kb, Additional file 1: Fig. S9B, C, D, E and Additional File 2: Table S6). Therefore, no common feature concerning the role of 3 mismatch small RNAs was observed between *T3* and the plasmid. Altogether, these results strongly suggest that the conversion efficiency of a sequence, not targeted in G1 but flanked by sequences targeted by the maternal piRNA pool depends, at least in part, on its length: in one generation, a low frequency of conversion can occur for sequences longer than 4 kb (i.e., *white* or *P(lArB)*), whereas the high frequency of conversion occurs for shorter sequences (i.e., plasmid sequence or *T3*).

### Conversion is restricted to sequences embedded within pre-existing piRNA clusters

The above results defined the conversion of loci surrounded on both sides by sequences targeted by maternal piRNAs. We then examined whether such conversion could also spread onto adjacent genomic sequences. Few 23–29-nt small RNAs flanking the insertion site of *P(lacW)* were detected that were not increasing between G1 and G4 (Additional file 1: Fig. S10A and B). The majority of them correspond to the transcribed strand of *Ago1* where the array of *P(lacW)* is inserted, suggesting that they were produced primarily by phasing without amplification [49]. The same analysis was performed on *CG17636*, the first gene on the *X* chromosome, close to *cluster 1A*. Few and unchanged 23–29-nt matching *CG17636* were identified between G1 and G4 (Additional file 1: Fig. S10C). Similar results were observed on endogenous homologous loci present in *P(lArB)* transgenes (Additional file 1: Fig. S11A, B, C). We conclude from these results that spreading of conversion from transgene sequences in *cis* as observed here and in earlier studies [43] or in *trans* outside the piRNA clusters is very limited suggesting the existence of a tight control that restricts piRNA cluster spreading and defines precisely their borders [44], like the transcription of genes flanking *cluster 42AB* or *cluster 80F* [10].

### *Cluster 1A* is a heterogeneous piRNA cluster

**Fig. 5:**
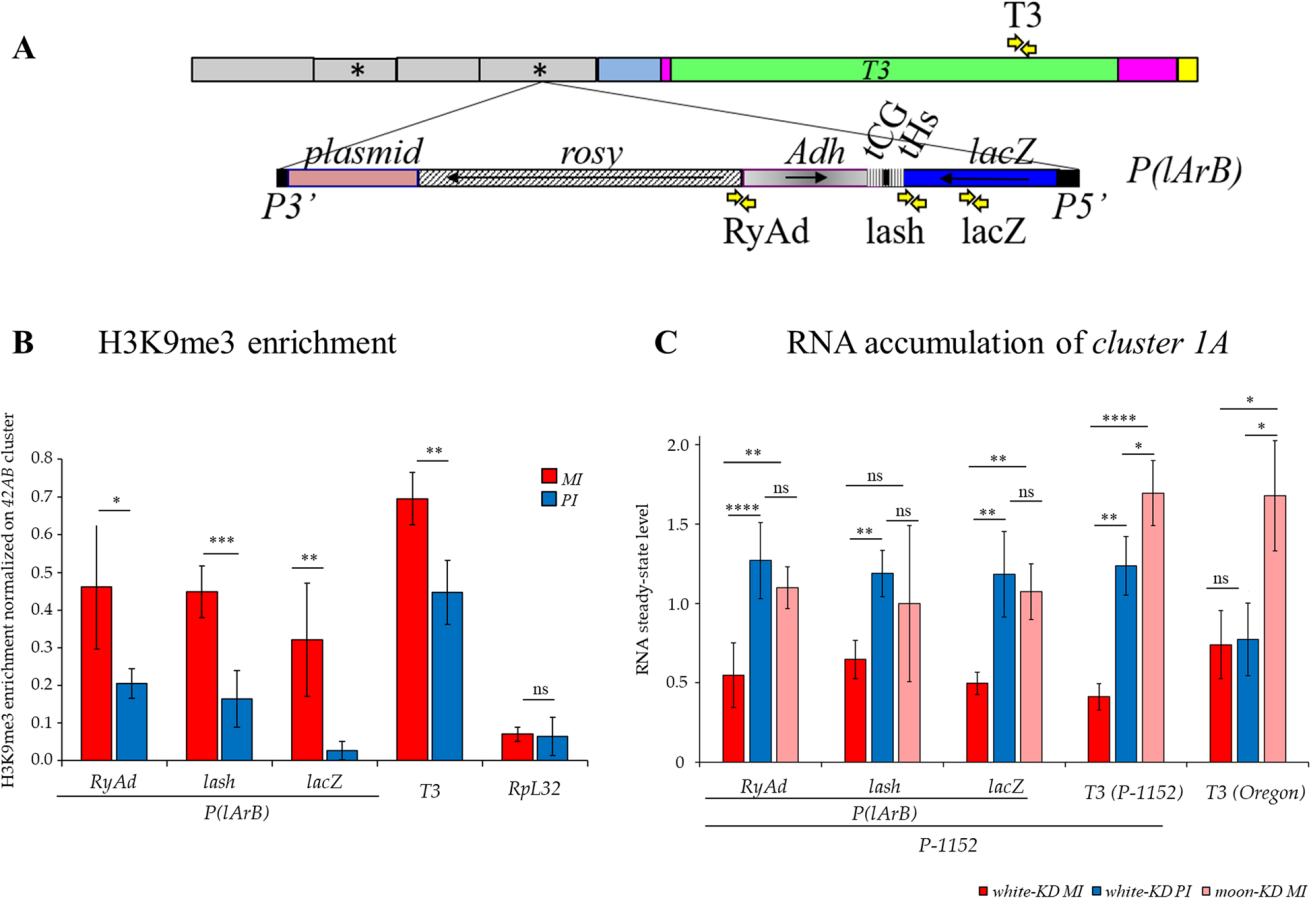
Chromatin state and steady-state transcription of *cluster 1A*. **A** Schematic representation of one of the subtelomeric repeats of *cluster 1A* of the *P-1152* strain, containing the *PlArB* transgenes (indicated by an asterisk). Yellow arrows indicate the position of qPCR primers. The *X* subtelomeric repeats (1.8 kb) and the *P(lArB)* (18 kb) are not drawn to scale. **B** H3K9me3 ovarian enrichment on three different regions of *P(lArB)* and of *T3* in maternal and paternal inheritance (*MI*, red; *PI*, blue) measured by ChIP-qPCR in G1. The signal was normalized to the *42AB* region highly enriched in H3K9me3 marks. ChIP experiments were performed on three independent biological samples. *P*-values were calculated using a bilateral *t*-test (*n* = 3). **B** Ovarian RNA accumulations of G1 *P(lArB)* and *T3* in *P-1152 (T3(P-1152))* and *T3* in *Oregon (T3(Oregon))* were measured by RT-qPCR in control KD (*whit*e-KD) and *moon-KD* and normalized to the expression of *RpL32* gene. *P*-values were calculated using a one-way ANOVA test followed by a Tukey HSD test (*n* = 3). ns, not significant. *P < 0.05, **P < 0.01, ***P < 0.001, ****P < 0.0001 (Additional file 2: Table S8)

Although *P(lArB)* and *T3* are located in the same *cluster 1A* of the *P-1152* strain, their piRNA profiles and kinetics of conversion when paternally inherited are different (Fig. 1E–G). To understand early molecular events occurring in G1, we first analyzed the ovarian heterochromatin throughout *cluster 1A*. Using chromatin immunoprecipitation followed by quantitative PCR (ChIP-qPCR, with primers shown in Fig. 5A, Additional file 1: Table S7), a high trimethylated Lysine 9 of Histone 3 (H3K9me3) enrichment was found on *P(lArB)*, when maternally inherited as compared to paternally inherited (Fig. 5B), confirming previous observations [5]. On *T3*, high H3K9me3 enrichment was observed in both *PI* and *MI*, with the overall level of H3K9me3 on *PI* being higher than on *P(lArB)* (Fig. 5B). Therefore, maternally inherited piRNAs can induce H3K9me3 enrichment on all sequences of *cluster 1A*. However, in *PI* G1, H3K9me3 enrichment is heterogeneous along the *1A* locus, from weak on *lacZ* to high on *T3*, consistent with their piRNA productions and silencing of *lacZ* and *pRFP-T3* piRNA sensors (Figs. 1, 2, and 3).

We next asked whether the chromatin variations observed in *P(lArB)* and *T3* and piRNAs synthesis were correlated with the RNA steady state of *cluster 1A*. For this, we analyzed the ovarian RNA accumulation across the locus by RT-qPCR experiments. Using the same primers (Fig. 5A), RNA accumulation of *P(lArB)* and *T3* in the *P-1152* strain were found higher in *PI* than in *MI* (see *white-KD* control in Fig. 5C). After four generations, differences between *PI* and *MI* were no longer detected, as the system reached an equilibrium similar to the maternal lineage (Fig. 1F, G, and Additional file 1: Fig. S12A). One explanation is that, in *MI*, high amounts of *P(lArB)* and *T3* piRNAs address the complementary *P(lArB)* and *T3* transcripts to the piRNA biogenesis inducing their degradation. In *PI*, low amounts of *P(lArB)* piRNAs (Fig. 1E, F) could instead prevent their recognition as piRNA precursors allowing accumulation of *P(lArB*) transcripts (Fig. 5C). Surprisingly, the high amount of *T3* piRNA in *PI* (Fig. 1E, G) was not correlated with low accumulation of *T3* containing transcripts (Fig. 5C). We therefore wondered if the presence of *P(lArB)* could affect *T3* RNA accumulation. As *cluster 1A* of *Oregon* was used to obtain *P(lArB)* insertion in the *P-1152* strain [50], we measured *T3* RNA accumulation in the *Oregon* strain (“*T3*(*Oregon*)”) and found unexpectedly that *T3* RNA steady state was unchanged between *MI* and *PI* contrary to *T3* in the vicinity of *P(lArB)* (“*T3(P-1152)*”) (Fig. 5C). The size and structure of germline piRNA cluster transcripts are still unknown; however, we assumed that chimeric transcripts can exist between different domains of piRNA clusters. Based on this assumption, in *T3(Oregon) PI*, chimeric transcripts containing *T3* and the other subtelomeric domains (*INV-4*, *T1*, *T2*, *T4*) can be targeted by piRNAs produced by the autosomal subtelomeric piRNA clusters. These transcripts are then directed to the piRNA degradation pathway with the same efficiency as in *MI* (Fig. 5C). In *T3(P-1152) PI*, the chimeric transcripts containing *T3* and subtelomeres are processed as described above leading to the production of piRNAs observed in Fig. 1G. In addition, the chimeric transcripts containing *T3* and *P(lArB)* can accumulate because they are not efficiently targeted by maternally inherited piRNAs that lack *T3* and *P(lArB)* (Fig. 5C).

We then tested if the non-canonical transcription specific of germline piRNA cluster was directly required for transcription of *P(lArB)* and *T3* in both genomic contexts (*T3(P-1152)* and *T3*(*Oregon*)). Germline knockdown of *moonshiner* (*moon-KD*, Additional file 1: Fig. S12B and C), involved heterochromatic transcription of dual-strand germline clusters [10], exhibited a clear increase of *P(lArB)* and *T3* RNA steady state compared to the control *white-KD MI* (Fig. 5C). These results indicate that c*luster 1A* transcription is Moon dependent, that these transcripts are funneled to the piRNA machinery, and that the presence of *P(lArB)* affects the RNA steady-state of *T3* in *T3(P-1152)* (Figs. 3E and 5C). One explanation is that transcriptional signals (initiation, termination) of *P(lArB)* transgenes might not be totally erased by the piRNA cluster chromatin and could therefore influence adjacent *T3* RNA accumulation.

**Fig. 6:**
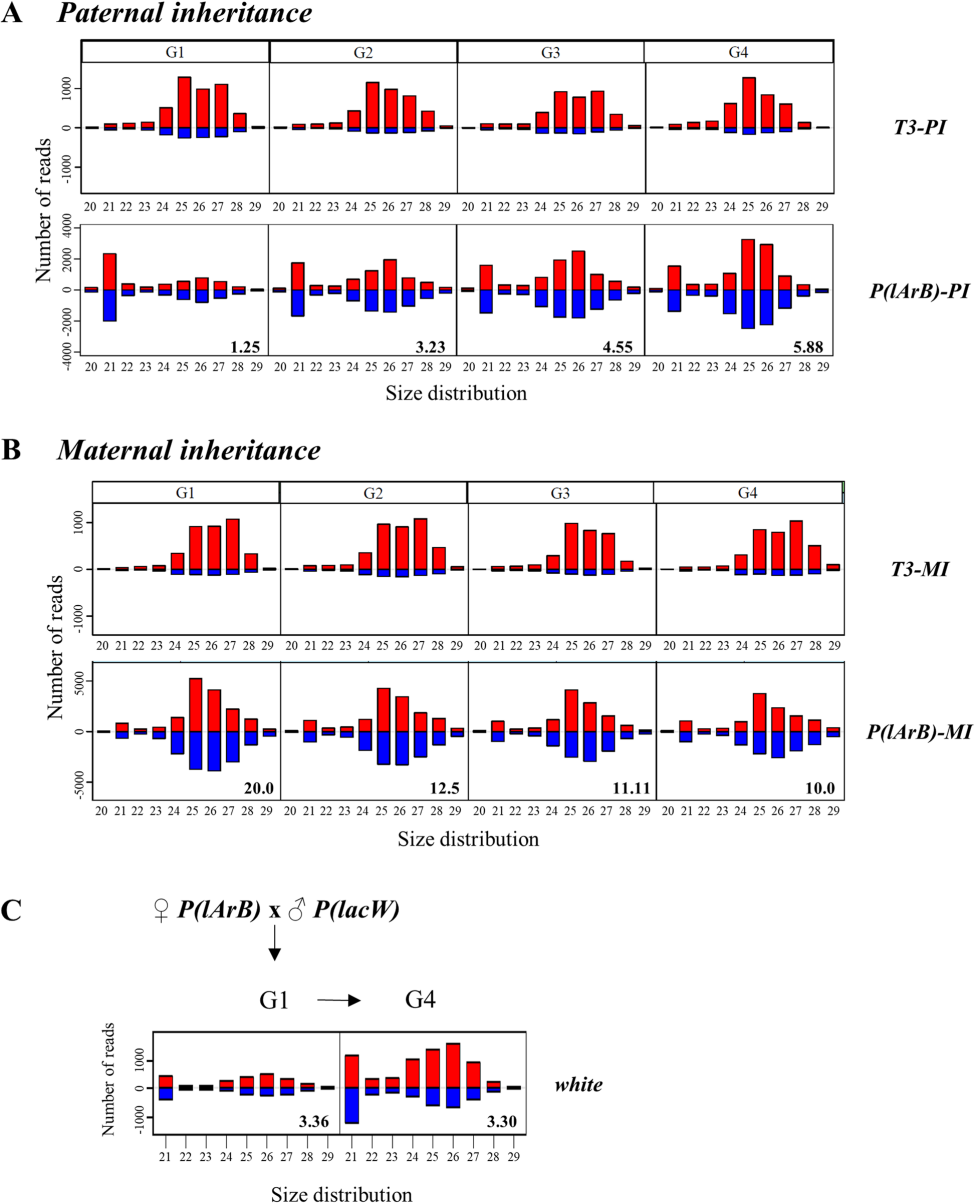
siRNAs and piRNAs abundance during conversion. Size distribution of ovarian small RNAs isolated from the *P(lArB)-PI*, subline D (**A**), or the *P(lArB)-MI* subline H (**B**) matching to *T3* and *P(lArB)* in G1, G2, G3, and G4. When paternally inherited, *P(lArB)* is converted for piRNA synthesis progressively across generations, while *T3* is converted from the first generation (G1). **C** Size distribution of small RNAs isolated in the G1 and G4 progenies of females containing *P(lArB)* crossed with males containing *P(lacW)* mapped on the *white* sequence. In this context, *white* gene is progressively converted (Fig. 4F). The ratio number of normalized 23–29-nt over 21 nt is indicated for the *P(lArB)* panel

According to dual-strand piRNA synthesis (Fig. 1E), accumulation of *P(lArB)* transcripts in G1 *PI* (Fig. 5C) could lead to the production of double-stranded RNAs that could be potentially processed into siRNAs in the absence of maternally inherited piRNAs. Consistent with a *bioRxiv* preprint from Luo et al. [51], we have questioned whether siRNAs were produced in parallel to piRNAs. We have compared the kinetics of occurrence of siRNAs and piRNAs during the *P(lArB)* conversion process in *PI* over the four generations (Fig. 1F). Indeed, a high amount of *P(lArB)* siRNAs is accumulated in G1 *PI* that persists across the first 4 generations, whereas piRNAs require the 4 generations to reach the plateau (Fig. 6A and Additional file 1: Fig. S13). The same profile of small RNA distribution was detected for all regions of the transgene in the *Canton* background and in the *w1118* genetic background (Additional file 1: Fig. S13A, B, and D). No such siRNA amount was found in the *MI* lineage, where *P(lArB)* was converted a long time ago (Fig. 6B and Additional file 1: Fig. S13C). Importantly, functional assays indicate that these transgenic siRNAs are not functional for the silencing of the *P(lacZ)* reporter (Fig. 1D, Additional file 2: Table S2). To complete this observation, we have also looked at siRNAs corresponding to the *whit*e gene when activated by *P(lArB)* (Fig. 4F). In this context, *white* siRNAs were produced from the first generation with a less spectacular abundance compared to *white* piRNAs, than in the case of *P(lArB)* G1 conversion, and that accumulate in G4 (Fig. 6C). Thus, the presence of siRNAs could precede the production of piRNAs, but this is not a general phenomenon. Their emergence can be also the result of accumulation in G1 *PI* of transcripts that are not targeted by maternal piRNAs and become the subtract of Dcr-2 endonuclease, in accordance with the fact that siRNAs were shown to be dispensable for germline piRNA cluster maintenance, silencing of piRNA sensor and paramutation [15, 34].

## Discussion

It is now well established that, although TEs are harmful to host genomes, they are also a source of evolution providing a large range of genetic opportunities, like new tissue gene-expression or novel genes [52]. Few of these events can be positively selected which has led to the notion of “molecular domestication” [53]. TEs have the capacity to transpose into or close to each other [54] forming highly enriched regions of TE fragments [55]. Some of these regions were shown to encode piRNAs involved in TE silencing in gonads of Metazoans [1, 3], the final domestication event granting TEs control of their own mobility. Accordingly, our understanding of the biochemistry and the genetics of piRNA biogenesis is well advanced [56]. In *Drosophila*, maternally inherited piRNAs can activate loci containing transgenes derived from *P* or *I* elements [12, 43, 57] and a euchromatic transgene can be activated for piRNA synthesis when its transcription is directed by the promoter of *HeT-A*, one of the telomeric TEs [58]. Early studies on hybrid dysgenesis revealed how newly horizontally transferred *P* elements could be maintained in *Drosophila* populations because of their capacity to insert into *cluster 1A* [11, 22–24, 30, 31, 34, 45]. This piRNA cluster can be lost under laboratory environment without affecting fly survival despite the fact that *P* could amplify and become stabilized most likely by new insertions into other piRNA clusters [26]. Analogous cases were found with a deletion of the *flamenco* locus that somatically derepressed the *ZAM* element leading to its *neo* insertion into a germline piRNA cluster [40] or after transgenesis of *Penelope* within *D. melanogaster* genome and its subsequent integration into a piRNA cluster [59]. These new insertions were *in fine* able to produce new piRNAs. However, how and at which rate newly inserted TE copy lose euchromatic identity to acquire piRNA cluster ones was unknown.

### Model of TE co-option by piRNA cluster: the secret of a successful horizontal transfer

**Fig. 7:**
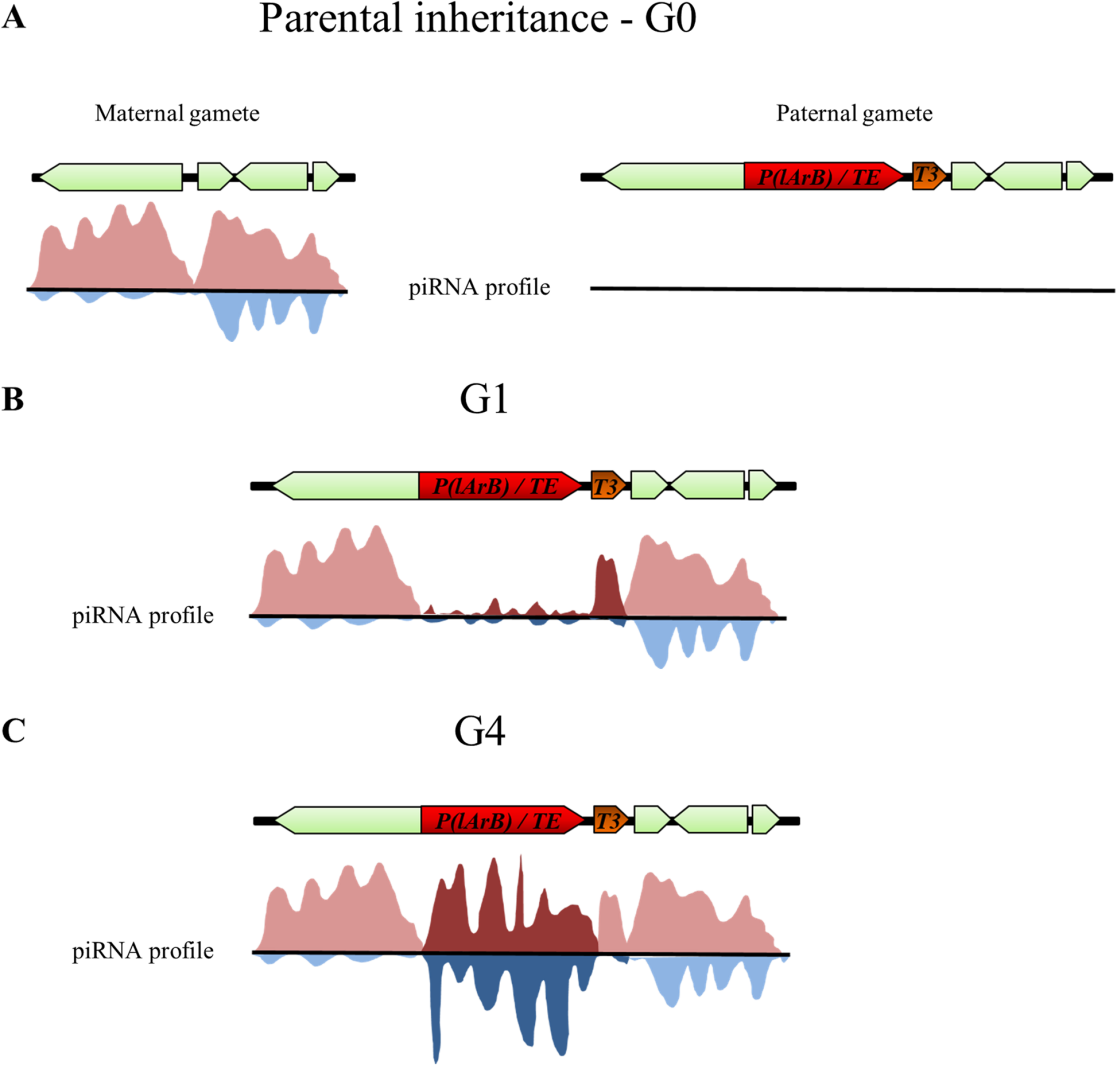
Model of sequence cooption revealing the existence of piRNA production heterogeneity within a piRNA cluster. **A** Maternally inherited piRNAs maintain active germline piRNA cluster at each generation (left), whereas paternal gametes transmit their DNA but no piRNAs to their progenies (right). **B** When a newly horizontally transferred TE inserts into a piRNA cluster, it is not efficiently targeted at the first generation (G1) by the maternal piRNA pool, even if the region is transcribed, resulting in a non-piRNA cluster conversion. This context is modeled in this study by the paternally inherited *P*-derived transgene, *P(lArB)* (red arrow), having a size closed to some autonomous TE but lacking complementary piRNAs. However, although paternally inherited and lacking homologous piRNAs, shorter sequences can be efficiently converted in G1, like *T3* (brown arrow). **C** Four generations (G4) can be sufficient to convert full-length TE, leading to new piRNA production. This conversion can lead to the synthesis of piRNAs with different piRNA profiles throughout the locus. Sense and antisense schematic piRNA profiles are extrapolated from this study, Marie et al., and Asif-Laidin et al. [26, 35]. Arrows symbolize TE fragments or repeats found in piRNA clusters, red and blue are for the sense and antisense piRNAs, and darker colors represent the “new” piRNAs, i.e., identified in a given generation (G1 or G4) and absent in the parents

Horizontal transfers of TEs been described in various organisms highlighting that such events can occur more frequently than originally thought [16]. However, the mechanisms and kinetics involved in taming these new invaders are not well understood. It has been modeled that upon a new insertion into a piRNA cluster, TE sequences are embedded in a locus that is targeted by maternal piRNAs. In our work, the paternally inherited *P(lArB)* transgenes inserted in *cluster 1A* or the *white* gene of *P(lacW)* activated by the *P-1152* strain recapitulate a TE, in length, inserted into a piRNA cluster in a naïve genome. Previously, it was shown in *D. simulans* that 20–40 generations are required to efficiently produce new piRNAs directed against the *P* element when monitored from the beginning of the invasion under environmental stress [60]. This result is in accordance with an evolutionary model that was based on several independent studies on the *P* element in *D. melanogaster* [61] and on the capacity of the *P* cytotype to take place across generations [62]. Other simulation studies have described three phases during TE invasion: a proliferative period followed by the occurrence and segregation of cluster insertions and finally fixation of cluster insertions [41]. The latter step requires reprogramming of piRNA cluster properties as shown for the *ZAM* element in *Drosophila* [40]. Once integrated, our results suggest that at each generation, egg chamber conversion will occur, and the TE copies will acquire stable piRNA cluster marks (Fig. 7) licensing non-canonical transcription [6, 9]. Once the conversion occurs, this epigenetic state is stably transmitted to the next generations. Altogether our work indicates that the long-term success of a new TE invasion depends principally on its capacity to insert into a piRNA cluster, because once inserted, any further transposition will be impeded. In addition, it has been proposed that some euchromatic copies of the new invaders might be targeted by the new piRNAs and contribute also to the production of piRNAs [63, 64]. A number of studies have also suggested that heterochromatic regions, like pericentromeric ones containing piRNA clusters, show a large accumulation of TEs as well as strong suppression of recombination [65, 66] limiting most likely deletions within inserted TE copies. Thus, while speculative but also based on our observations, some of the successful TE HT might also drive positive selection toward non-autonomous deleted copies, instead of full-length copies, inserted into piRNA clusters. Moreover, the efficiency of conversion which seems to depend on the size of the sequences (Figs. 1 and 4) might be interpreted as a safeguard that might exist to define piRNA cluster borders, avoiding deleterious propagation on flanking euchromatic regions. Future works will be necessary to identify factors involved in defining piRNA cluster borders and canonical *vs* non-canonical piRNA-related transcription. Finally, this work highlights that maternal piRNA legacy is a fundamental prerequisite for genome stability that has to be established and then inherited at each generation in order to maintain active maternal and paternal piRNA cluster alleles.

### Non-allelic paramutations between piRNA clusters occur at each generation to maintain genome stability

Because piRNA clusters are composed for the most part of fragments of TEs, it is difficult to conduct an extensive analysis of their dynamics. We concentrated our effort on *cluster 1A*, because strains devoid of this locus exist, *T3* is restricted to the *X* subtelomere and it is long enough to conduct simple molecular analysis [26]. Assuming that *T3* can recapitulate to some extent the properties of the whole native *cluster 1A*, our analysis indicates that maternally inherited piRNAs targeting redundant subtelomeric sequences, such as *INV-4*, *T1*, *T2*, and *T4*, produced by the autosomal subtelomeres, are able to epigenetically convert paternally inherited *T3* sequence from the first generation. Such epigenetic conversion, dependent on maternal piRNAs able to convert allelic and non-allelic loci, is known as paramutation [12, 15]. It might be the keystone to convert paternal loci of germline piRNA clusters at each generation. In addition, studies of piRNA cluster through several *Drosophila* species identified that piRNA clusters are genomic regions evolving rapidly due, in part, to recurrent chromosomal rearrangements [63, 67]. Once again, non-allelic paramutation appears to be an efficient mechanism to maintain the mandatory pool of piRNA producers activated by maternally inherited piRNAs produced by any loci to maintain genome integrity. These results reveal the selection forces that might exist to preserve hundreds of piRNA clusters with redundant sequences that secure efficient TE control at each generation (Fig. 7).

### Germline piRNA clusters can be heterogeneous entities

In *Drosophila*, germline piRNA clusters can be dual-strand or uni-strand clusters. So far, most descriptions of piRNA clusters have considered these loci of several dozens of kb as epigenetically and functionally homogeneous regions. The *cluster 42AB*, the largest dual-strand cluster, is H3K9me3 enriched that recruits Rhi, which binds to Deadlock interacting with Moon allowing initiation of transcription on both strands. On the contrary, *cluster 20A* is a uni-strand cluster whose transcription is Moon-independent and mildly enriched in H3K9me3 [6, 10]. Here, we have dissected the molecular and genetic properties of *cluster 1A* by focusing on two domains (*P(lArB)* and *T3*) which are separated by about 500 bp from each other. Although they are both H3K9me3 enriched and Moon dependent, our results indicate that they differ by the ratio of sense *vs* antisense piRNA distribution. *P(lArB)* results in a symmetric dual-strand region, while *T3* is an asymmetric dual-strand domain (Fig. 7). This observation might indicate that piRNA clusters are not strictly epigenetically determined but may involve genetic properties, which are not completely erased by the piRNA cluster chromatin. In addition, they differ by the kinetics of conversion when they are paternally inherited in the absence of maternal complementary piRNA inheritance. These results have revealed an unexpected feature for piRNA clusters like *cluster 1A* in that they can be rather heterogeneous (Fig. 7). Is this property unique to c*luster 1A*? In the light of our results and inspection of that of others, *cluster* *80F* displays also a heterogeneous piRNA profile distribution being a dual-strand cluster for the most part of the locus and a uni-strand/asymmetric cluster in the 3′ region [6, 10, 47]. Furthermore, in the absence of *moon*, germline *42AB* and *38C1* dual-strand piRNA clusters display an increasing number of piRNAs localized to the 3′ region of *cluster 42AB* and to the whole cluster *38C1* [10]. Therefore, it would be interesting to design piRNA sensors for different parts of these clusters to functionally test their silencing in this context. RT-qPCR data revealed that Moon is required for the transcription of *P(lArB)* and *T3* with an increase of about twofold of RNA accumulation for *P(lArB)* and *T3*. Finally, our data suggest that the primary properties of a sequence to become part of a piRNA cluster, in the absence of complementary maternally inherited piRNAs, could depend on the genomic environment, on the composition of chromatin but also on intrinsic features of sequences, for instance, transcriptional signals. Recently, Kipferl, a DNA-binding protein, has been shown to recruit Rhi at piRNA clusters through specific sequences and is required for some functional piRNA clusters [68]. This underlines a genetic and an epigenetic part for piRNA cluster determination. Therefore, by dissecting *cluster 1A*, we uncovered heterogeneity within large piRNA clusters revealing that piRNA cluster loci are more intricate regions than originally thought.

## Conclusions

Using transgenic tools inserted into piRNA clusters, piRNA sensors, *cluster 1A*, and the fact that no paternally inherited piRNAs are transmitted to the progenies, we could address the dynamics of production of new piRNAs from sequences located within piRNA cluster but absent from the maternal piRNA repertoire. This situation is encountered in the case of horizontal transfer of TEs. During our analysis on *cluster 1A* as a model of horizontal transfer events, we identified that this cluster is able to respond within 4 generations to co-opt a sequence of about a TE size, not belonging to the maternal piRNA repertoire, whose transcription become *moon* dependent leading to the synthesis of new piRNAs. Our study also identified that a piRNA cluster can be heterogeneous, as different domains can display different piRNA profiles leading to symmetric next to asymmetric dual-strand piRNA cluster regions. As piRNA clusters contain TE fragments, these results lead us to suggest that some of these fragments might have an influence on the piRNA cluster biology leading to the heterogeneity that we observed.

## Methods

### *Drosophila* strains

*P-1152* (FBti0005700) strain carries two enhancer trap *P(lArB)* transgenes that contain an in-frame translational fusion of the *E. coli lacZ* gene to the second exon of the *P* transposase gene [50]. They are inserted in one of the subtelomeric repeats of the *X* chromosome that is one of the piRNA clusters, we named *cluster 1A* (Fig. 1A, B) [32, 45, 50]. This strain also contains the autosomic subtelomeric repeats on the *3R* chromosome, that is a piRNA cluster (*cluster 100F*) [26] and is mutant for the *white* gene (*w*^*c*^ allele). The *Canton* strain is devoid of *P* element and *cluster 1A* [26], hereafter referred to as a *Δ-1A* strain. The *Canton* line used is marked by a *yellow* allele affecting body color pigmentation and is derived from the classical laboratory strain. The *BX2* strain contains seven *P(lacW)* transgenes (FBtp0000204) tandemly inserted in euchromatin on the second chromosome [69]. *P(RS3)* is a *P-FRT-white* transgene (FBtp0001534) inserted in the subtelomere of the *3R* chromosomal arm (*cluster 100F*) [70]. The *lacZ* piRNA sensor (*BQ16*, FBtp0000154) carries a euchromatic *P-lacZ* fusion enhancer trap transgene strongly expressing *lacZ* in the germline [71]. When *P-1152* females are crossed by *lacZ* piRNA sensor males, *lacZ* is strongly repressed in the female germline [32, 34]. *Oregon* strain is a classical laboratory strain devoid of the *P* element with the *X* (*cluster 1A*), *2R* (*cluster 60F*), and *3R* (*cluster 100F*) subtelomeric piRNA clusters [26]. Its *X* chromosome was present in the strain used to generate the *P-1152* strain [50].

The *pRFP-T3* transgenic strain carries an insertion of the *RFP* reporter gene transcriptionally fused to the *T3* domain. It was obtained after transformation into the *w*^*1118*^ strain, a *Δ-1A* strain. This *T3* piRNA sensor was obtained by cloning the PCR-amplified *RFP* gene (using primers: 5′-AGGTACCATGCCCAAGAAGAAGCGCAAGGTGGCCTCCTCCGAGGACGTCATCAAG-3′ and 5′-ATCTAGATTAGGCGCCGGTGGAGTGGC-3′) into the *pUASp* plasmid [72] digested with *Kpn*I and *Xba*I, followed by inserting into the *Xba*I site, the PCR amplified *T3* sequence (primers used: 5′-AATCTAGACCCAGCAAATTTATGGATAAAC-3′ and 5′-ATTCTAGACCTAATTTTTGGCAAAGTTGTAC-3′). Female germline expression of *pRFP-T3* is driven by the *PBac{w[*+ *mW.hs]* = *GreenEye.nosGAL4}* (FBtp0056000) transgene. Germline-specific knockdowns (KD) were performed by crossing short hairpin or RNA interference transgenes [10, 47, 73] directed against genes under the UAS sequence with the Gal4 maternal driver under the promoter of the *nos* gene (FBtp0056000 transgene). Additional file 2: Table S1 summarizes the characteristics of each strain used in this study.

### Experimental conditions and measurements of *lacZ* and *RFP* silencing

All crosses were performed at 25 °C. The *y* and *w* marker genes present on the *Canton* (*Δ-1A*) and *P-1152* strains, respectively, were used to control the subtelomere segregation in successive generations as well as to identify rare recombination events. We generated hemizygous females for the subtelomeric *P(lArB)* transgenes with a maternal (*MI*) or paternal (*PI*) inheritance (Fig. 1C) that were then crossed with *Canton* males in successive generations. In parallel, silencing capacities of *MI* and *PI* lines were measured using a germline functional assay at each generation for four independent sublines by crossing hemizygous females at each generation with males containing the *lacZ* piRNA sensor (Additional file 1: Fig. S2B). Ovarian *lacZ* expressions were assayed using X-gal staining and the repression was quantified as previously described by determining the percentage of egg chambers with no *lacZ* expression in the germline [34]. Ovarian RFP expressions were assayed by fixing ovaries in 5% formaldehyde during 6 min, washing in 1X PBS (3 times), and overnight incubating in PBS/DAPI. The ovaries were then washed 3 times in PBS 1X and then spread on a slide with a mounting medium (80% glycerol in PBS, 4% propylgalate) [74]. The silencing capacity of subtelomeric *T3* piRNAs was quantified by determining the percentage of repressed egg chambers carrying a *pRFP-T3* reporter transgene. The quantification was restricted to stages 8–10 of egg chambers where the RFP expression was intense and reproducible. Images were acquired with an Axio-ApoTome (Zeiss) and ZEN2 software.

### Ovarian small RNA sequencing and analysis

Total RNA extraction, small RNA libraries preparation, and sequencing were performed as previously described [15]. Sequence reads in fastq format were trimmed from the adapter sequence 5′-TGGAATTCTCGGGTGCCAAG-3′ and matched the reference sequences using Bowtie [75]. Annotation of small RNA libraries is described in Additional file 2: Table S4. Small RNAs matching the reference sequences with 0 mismatches were retained for subsequent analysis, except when notified. Sequence length distributions and small RNA mapping were generated from bowtie alignments using Python and R (www.r-project.org/) scripts, which were wrapped and run in a Galaxy instance publicly available at http://mississippi.sorbonne-universite.fr/. Tools used in this study may be downloaded from this Galaxy instance. For library comparisons, read counts were normalized (normalization factor) relative to the number of sequence reads aligning to the *D. melanogaster* genome but not to miscRNAs (including rRNA and snoRNA) or tRNAs [12]. Similar results were obtained, when tested by normalizing the read counts to one million miRNA reads or to one million *D. melanogaster* reads (Additional file 1: Fig. S14A, Table S4A). Furthermore, no global differences among libraries were identified when comparing the ratio of 23–29-nt reads over the miRNA reads between several libraries (Additional file 1: Fig. S14B, Additional file 2: Table S4).

### RT-qPCR

For each sample, 2 μg of total RNA was treated with DNase (Fermentas). One microgram of DNase-treated RNA was used for reverse transcription (RT) using random hexamer primers (Fermentas). Real-time qPCR was performed on triplicates of each sample using primers referred to in Additional file 2: Table S7. The *RpL32* gene was used as a reference. The same series of dilution of a mix of different RT preparations was used to normalize the quantity of transcripts in all RT preparations leading to standard quantity (Sq) values. Variations between technical triplicates were very low when compared to variations between biological replicates. The mean of the three technical replicates was then systematically used (meanSq). For each biological sample, we calculated the ratio meanSq(*gene*)/meanSq(*Reference Gene*) to normalize the transcript quantity. Then, the mean of each sample ratio was used to compare the two conditions.

### ChIP-qPCR

For each sample, 50 to 100 pairs of ovaries from 2- to 3-day-old females were manually dissected in cold 1X PBS, crossed-linked with 1.8% formaldehyde, quenched with glycine, washed with 1X PBS, and collected by centrifugation. Pellets were flash-frozen in liquid nitrogen. The ovaries were ground with a pestle and lysed in ChIP lysis buffer (NaCl 100 mM, Tris pH8 50 mM, EDTA 5 mM, 1% SDS). Lysates were sonicated using Bioruptor (Bioruptor Standard Diagenode) three times during 15 min (30 s ON, 30 s OFF) and cleared by centrifugation. Five percent of cleared lysate was set aside to serve as input samples, and the remainder was divided in two equal portions and incubated at 4 °C with antibodies overnight under gentle rotation (anti-H3K9me3 from Active Motif Cat#39161, negative IgG control from Diagenode Low Cell ChIP-kit Cat#803-015). Magnetic beads coupled to G protein (Dynabeads Protein G, Invitrogen Cat#10003D) were washed two times in low salt wash buffer (0.1% SDS, 1% Triton X-100, EDTA 2 mM, Tris pH8 20 mM, NaCl 150 mM), transferred into immunoprecipitated lysate and incubated at 4 °C during 1 h under gentle rotation. The beads were washed two times in low-salt wash buffer, one time in high-salt wash buffer (0.1% SDS, 1% Triton X-100, EDTA 2 mM, Tris pH8 20 mM, NaCl 500 mM), two times in LiCl wash buffer (LiCl 0.25 M, 1% NP-40, 1% Na deoxycholate, EDTA 1 mM, Tris pH8 10 mM), and two times in TE buffer (Tris pH8 10 mM, EDTA 1 mM). Decrosslinking, elution, and DNA purification were performed using an IPure kit (Diagenode Cat# C03010015).

Real-time PCRs were performed on duplicates for each biological sample leading to cycle threshold (Ct) values. Variations between technical duplicates were very low compared to variations between biological replicates. The mean of the two technical replicates was then systematically used (meanCt). For each sample, the IP fraction was normalized beside input to take account of sample preparation difference as follows: ΔCt [normalized ChIP] = (meanCt [ChIP] − (meanCt [Input] − Log2 (Input dilution factor))) where meanCt [ChIP] is the Ct value measure for immunoprecipitated samples, and meanCt [Input] is the Ct value measure for input and input dilution factor corresponds to the chromatin fraction set aside for input (in this experiment, 5% of chromatin fraction was set aside, thus input dilution factor was 20). To confirm specific antibody signals compared to the negative control, fold enrichment was calculated for each sample as follows: fold enrichment = 2^(−ΔCt [normalized ChIP] − ΔCt [normalized NS]^. Fold enrichment values are then normalized on the *42AB* region, used as a control region.

## Supplementary Information

**Additional file 1: Fig. S1.** Mapping of complementary maternally inherited piRNAs on a piRNA cluster locus. **Fig. S2.** Parental strains and experimental schemes. **Fig. S3.** Concomitant conversion of all the regions of the *P* transgene in the *MI* H and *PI* B sublines. **Fig. S4.** Conversion of *cluster1A *in the *Δ-1Aw*^*1118*^ background. **Fig. S5.** Conversion of the *P* transgene inserted in the autosomal *cluster100F*. **Fig. S6.*** P*-derived sequences of *P* are able to repress ovarian expression of *pRFP*. **Fig. S7.** Ovarian *lacZ* silencing induced by *P* is impaired by germline knockdown of genes involved in piRNA biology. **Fig. S8.** Crosses used for the conversion of the *P* transgenes by *P* or *P*. **Fig. S9.** Study of composition of converted sequences. **Fig. S10.** No *cis-*conversion of flanking regions of the *P* transgene clusters or of *cluster 1A*. **Fig. S11.** No *trans*-conversion of endogenous homologous sequences. **Fig. S12.** RT-qPCR experiments of *P* and *T3 *in* P-1152*. **Fig. S13.** siRNAs and piRNAs abundance during conversion of *cluster 1A*. **Fig. S14.** Comparison of methods for small RNA libraries normalizations.

**Additional file 2: Table S1.** List of *Drosophila* strains. **Table S2.** Ovarian *lacZ* repression for the maternally and paternally inherited* P*transgenes. **Table S3.** Normalized number of unique mappers of 23 to 29 reads in the 3 sublines sequenced. **Table S4.** Annotation of small RNA libraries. **Table S5.** Percentage of dinucleotides content. **Table S6.** Number of 21 ntand 23-29 ntreads with 0 or 3 mismatches mapping to *P*, *T3*, *white* and plasmid. **Table S7.** Primers used in this study. **Table S8.** *P-*values calculated for the H3K9me3 ovarian enrichment by ChIP-qPCR.

## Data Availability

Small RNA sequencing data have been deposited at the European Nucleotide Archive (ENA) under accession numbers PRJEB11491 for GRH12 (https://www.ncbi.nlm.nih.gov/sra/PRJEB11491, [15, 76]), PRJEB19350 for GRH129 and GRH133 (https://www.ncbi.nlm.nih.gov/sra/PRJEB19350, [26, 77]), PRJNA899103 (https://www.ncbi.nlm.nih.gov/sra/PRJNA899103 [78]), and PRJNA935551 (https://www.ncbi.nlm.nih.gov/sra/PRJNA935551 [79]).

## Abbreviations

TE: Transposable element
piRNA: PIWI-interacting RNA
HT: Horizontal transfer
TAS: Telomeric-associated sequences
bp: Base pair
nt: Nucleotide
RT: Reverse transcriptase
qPCR: Quantitative PCR
ChIP: Chromatin immunoprecipitation
RFP: Red fluorescent protein
PI: Paternal inheritance
MI: Maternal inheritance
nos: Nanos
boot: Bootlegger
moon: Moonshiner
Rhi: Rhino
Del: Deadlock
Cuff: Cutoff
RDC complex: Rhi-Del-Cuff complex
LTR: Long terminal repeat
*D*.: *Drosophila*
