# Supplementary material for: Modeling early germline immunization after horizontal transfer of transposable elements reveals internal piRNA cluster heterogeneity
Source: BMC Biol. 2023 May 24;21:117. doi: 10.1186/s12915-023-01616-z (PMC10210503; doi:10.1186/s12915-023-01616-z)
Supplement: Supplementary file 1 — Additional file 1: Fig. S1. Mapping of complementary maternally inherited piRNAs on a piRNA cluster locus. Fig. S2. Parental strains and experimental schemes. Fig. S3. Concomitant conversion of all the regions of the P transgene in the MI H and PI B sublines. Fig. S4. Conversion of cluster1A in the Δ-1Aw1118 background. Fig. S5. Conversion of the P transgene inserted in the autosomal cluster100F. Fig. S6. P-derived sequences of P are able to repress ovarian expression of pRFP. Fig. S7. Ovarian lacZ silencing induced by P is impaired by germline knockdown of genes involved in piRNA biology. Fig. S8. Crosses used for the conversion of the P transgenes by P or P. Fig. S9. Study of composition of converted sequences. Fig. S10. No cis-conversion of flanking regions of the P transgene clusters or of cluster 1A. Fig. S11. No trans-conversion of endogenous homologous sequences. Fig. S12. RT-qPCR experiments of P and T3 in P-1152. Fig. S13. siRNAs and piRNAs abundance during conversion of cluster 1A. Fig. S14. Comparison of methods for small RNA libraries normalizations. [file 12915_2023_1616_MOESM1_ESM.pdf]

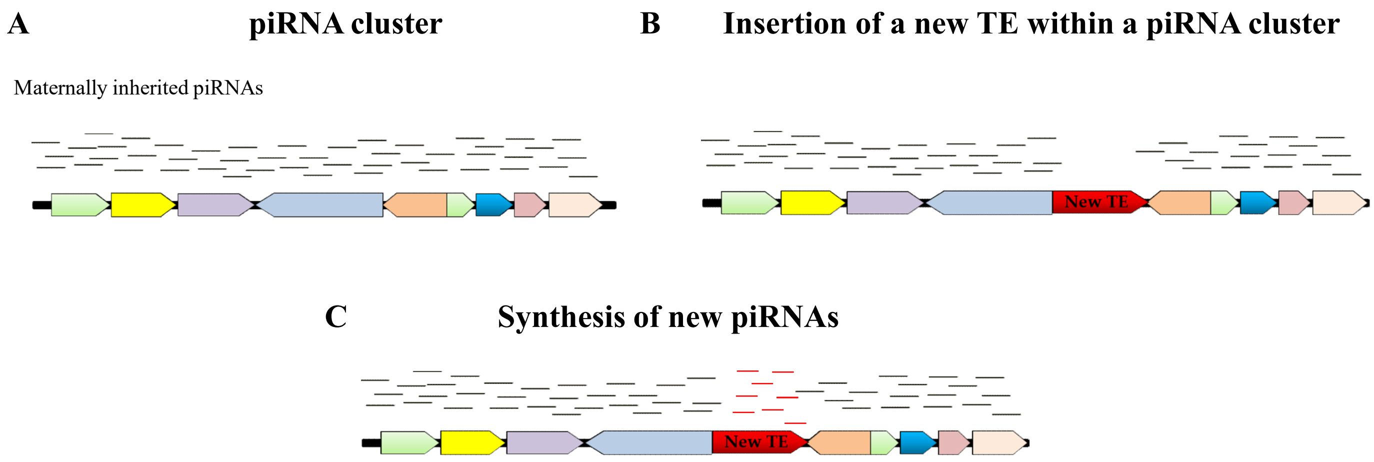

Figure S1. **Mapping of complementary maternally inherited piRNAs on a piRNA cluster locus**. **A.** The numerous piRNA clusters are enriched in TEs, that are repeated sequences. Maternal piRNAs (black lines) produced from active piRNA clusters are targeting complementary sequences at each generation. This maternal inheritance is necessary to define piRNA clusters at each generation whether the producer locus is allelic or not thanks to redundancy between clusters. **B.** As no paternal piRNAs are inherited, if a piRNA cluster, that contains a domain absent from the maternal clusters, is exclusively paternally inherited, this domain will be surrounded by sequences targeted by maternal piRNAs in the progenies. This situation is also encountered when a newly horizontally transferred TE inserts into a piRNA cluster (red arrow). **C.** This copy will be eventually co-opted by the locus leading to the synthesis of new piRNAs complementary to the new TE (red lines). The precise kinetics of this phenomenon was unknown and was questioned in this study.

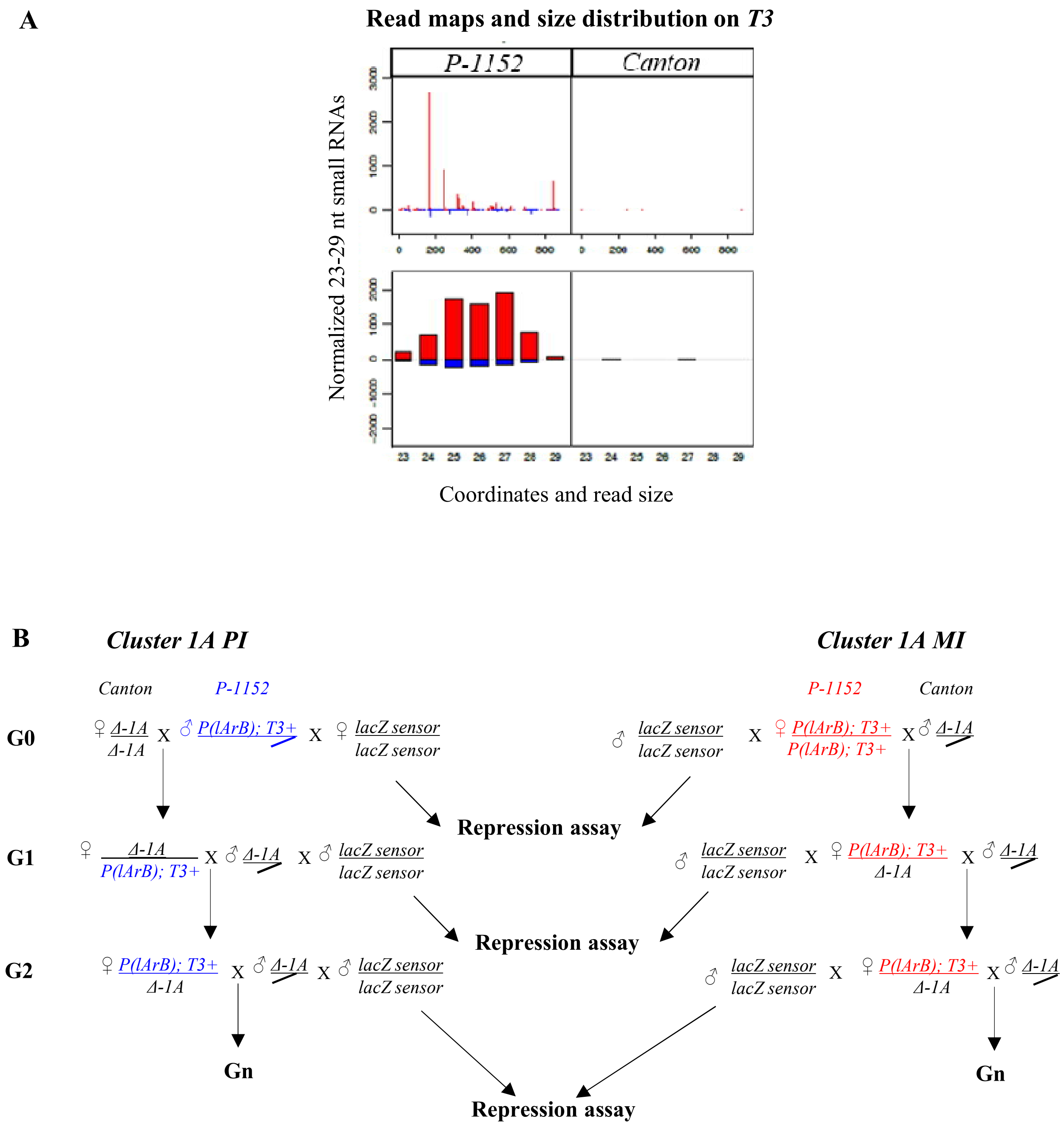

Figure S2. Parental strains and experimental schemes. A. Normalized 23-29 nt small RNAs extracted from ovaries of the *P-1152* and the *Δ-1 Canton* strains were mapped on the *T3* sequences (red are sense and blue are antisense reads, read maps above and size distribution below). **B.** Experimental schemes of the repression assays in *P(lArB) PI* and *MI* lineages. Ovarian *lacZ* repression was assayed by a X-gal staining on both lineages by crossing *P-1152* individuals at each generation with flies carrying the euchromatic *lacZ* sensor.

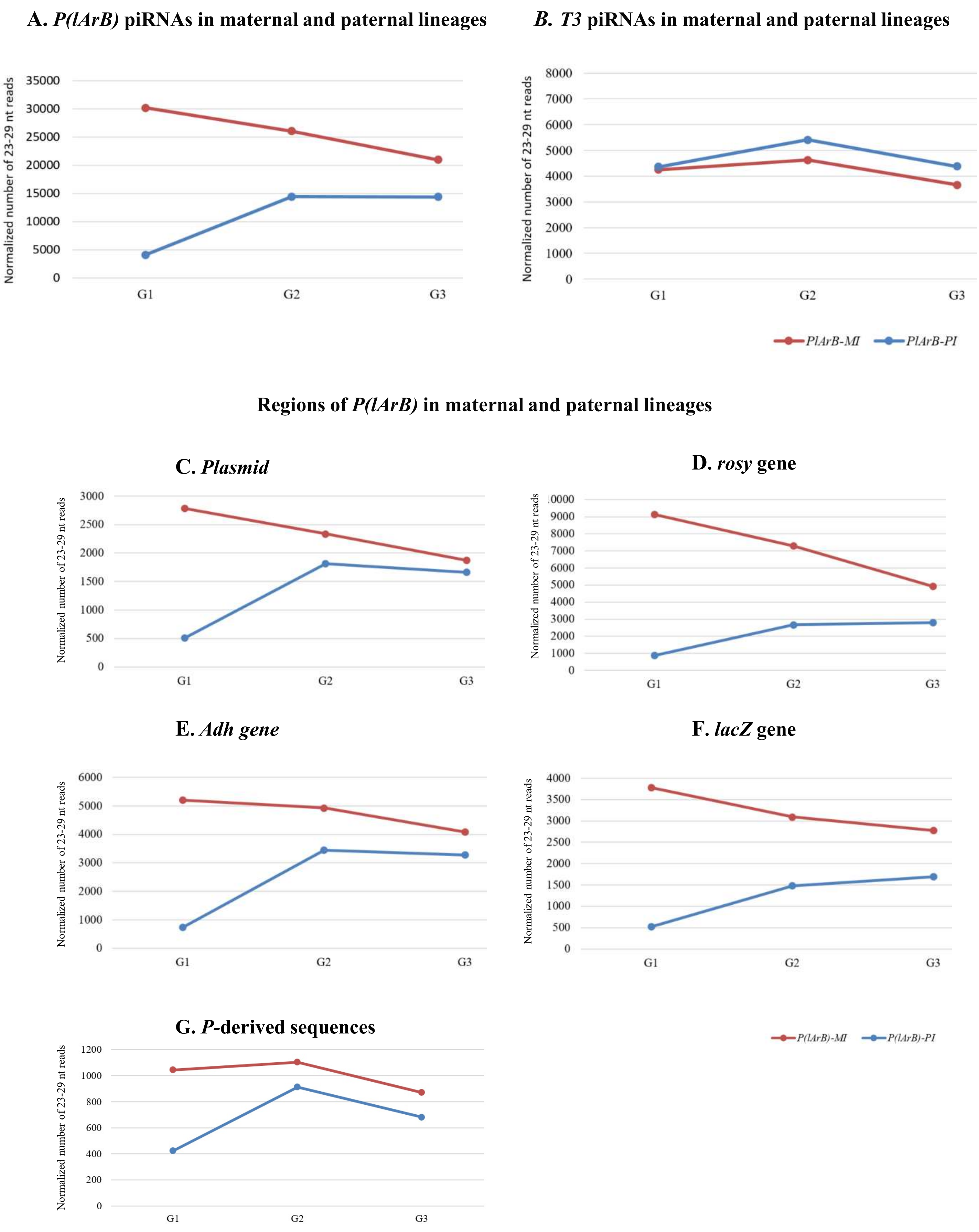

Figure S3. Concomitant conversion of all the regions of the *P(lArB)* transgene in the *MI* H and *PI* B sublines. Normalized 23-29 nt reads mapping to *P(lArB)* (**A**), *T3* (**B**) and the regions of the *P(lArB)* transgene: plasmid (**C**), *rosy* (**D**), *Adh* (**E**), *lacZ* (**F**) genes and *P*-derived sequences (**G**). See Additional File 1: Fig. S2B for crosses information.

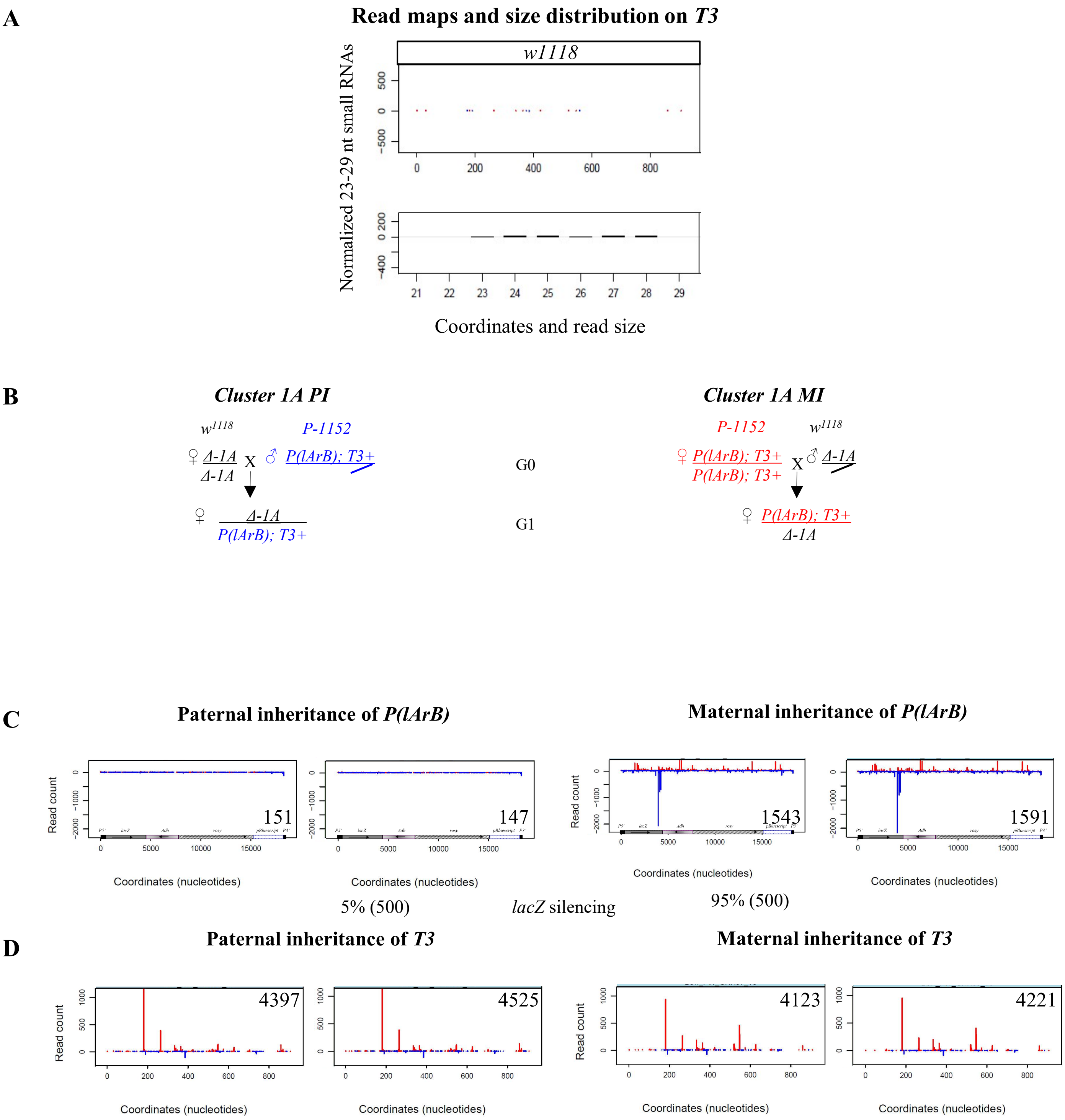

Figure S4. Conversion of *cluster 1A* in the *Δ-1A w^1118^* background. A. Normalized 23-29 nt small RNAs extracted from ovaries of the *w1118* strain were mapped on the *T3* sequences (read maps above and size distribution below). **B.** Reciprocal crosses leading to *MI* and *PI* inherited *cluster 1A* carrying the *P(lArB)* transgenes and the *T3* domain. The name of each strain is indicated above the genotype. The indicated genotypes correspond to the *X* alleles. The *w^1118^* strain is lacking *cluster 1A (Δ-1A)* but contains the *60F* and *100F* autosomal subtelomeric piRNA clusters. **C** and **D**. Normalized 23-29 nt small RNAs were mapped on the *P(lArB)* (**C**) and *T3* sequences (**D**) in G1. Results on duplicates are shown for each analysis, indicating no or very few differences between duplicates**.** Numbers in each panel represent the density of normalized 23-29 nt reads per kb in each condition (reads/kb). Contrary to the *P(lArB)* transgenes, one generation is sufficient to convert the *T3* domain for piRNA production.

Asif-Laidin et al.,

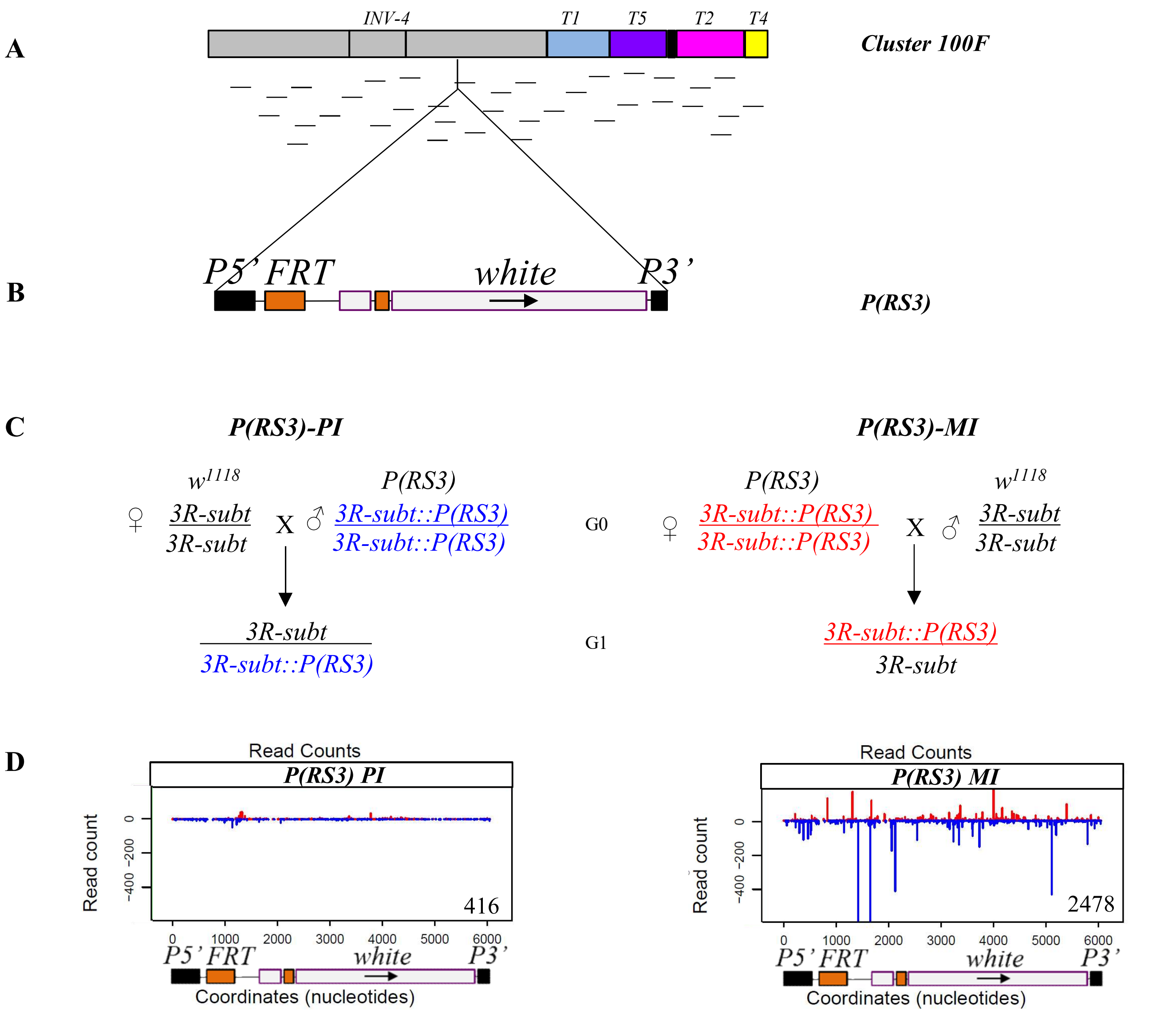

Figure S5. Conversion of the *P(RS3)* transgene inserted in the autosomal *cluster 100F*. A. One of the subtelomeric repeats of *cluster 100F* (right arm of the third chromosome, 3R) is shown (0.98 kb). Each repeat is composed of several domains common with *clusters 1A* and *100F* (*INV-4*, *T1*, *T2* and *T4)*, and a domain specific to the autosomal subtelomeres (*T5*). Small black lines symbolize the maternal piRNAs produced by *cluster 100F*. **B.** Structure of the *P(RS3)* transgene inserted in *cluster 100F*. It is encoding the *mini-white* gene (4.1 kb), interrupted by a *FRT* sequence. The two regions are not drawn to scale. **C**. Reciprocal crosses performed to obtain *PI* and *MI P(RS3)*. *"3R-subt::P(RS3)"* and *"3R-subt"*stand for the *3R* subtelomeric locus (*cluster 100F*) with or without an insertion of *P(RS3)*, respectively. The name of each strain is indicated above the genotype. **D.** Normalized ovarian 23-29 nt small RNAs mapping to *P(RS3)* transgene in G1. The experiment shows that *100F* piRNAs are unable to convert *P(RS3)* at the first generation. Numbers in each panel represent the density of normalized 23-29 nt reads per kb (reads/kb).

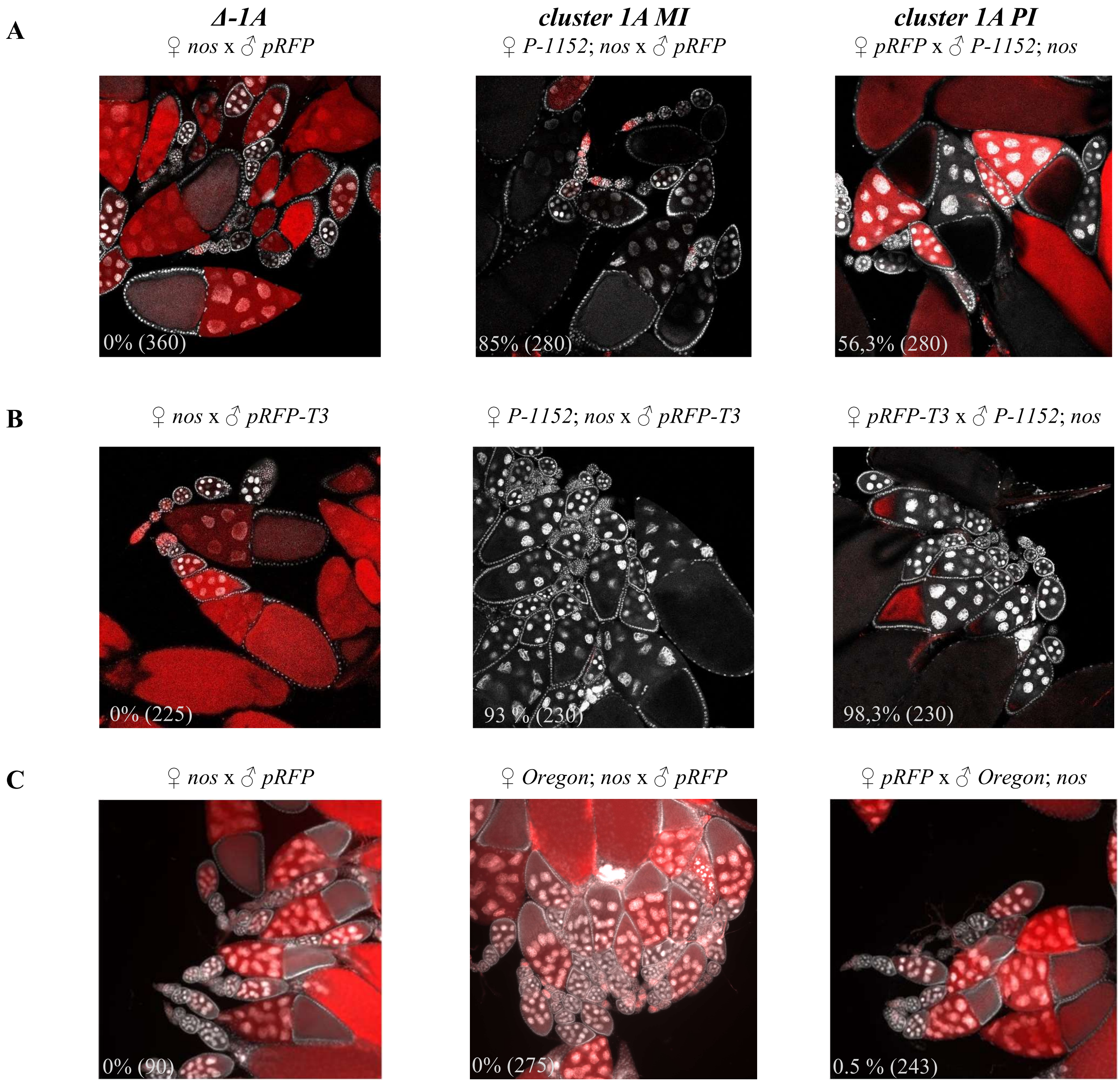

Figure S6. *P*-derived sequences of *P(lArB)* are able to repress ovarian expression of *pRFP*. A. The *P(lArB)* transgenes inserted into *cluster 1A* of *P-1152* repress the expression of the *pRFP*. **B.** Ovarian expression of the *pRFP-T3* transgene is repressed by the *P(lArB)* and *T3* inserted in *cluster 1A* of the *P-1152* strain. The *pRFP-T3* repression observed when *P-1152* is paternally inherited (third column) corresponds most likely to repression induced by *T3* and *P*-derived sequences. **C.** Ovarian expression of *pRFP* transgene is not affected by *cluster 1A* of the *Oregon* strain, that is devoid of *P*-derived sequences. Parental crosses are indicated above images. The percentage of repressed egg chambers are indicated in each panel.

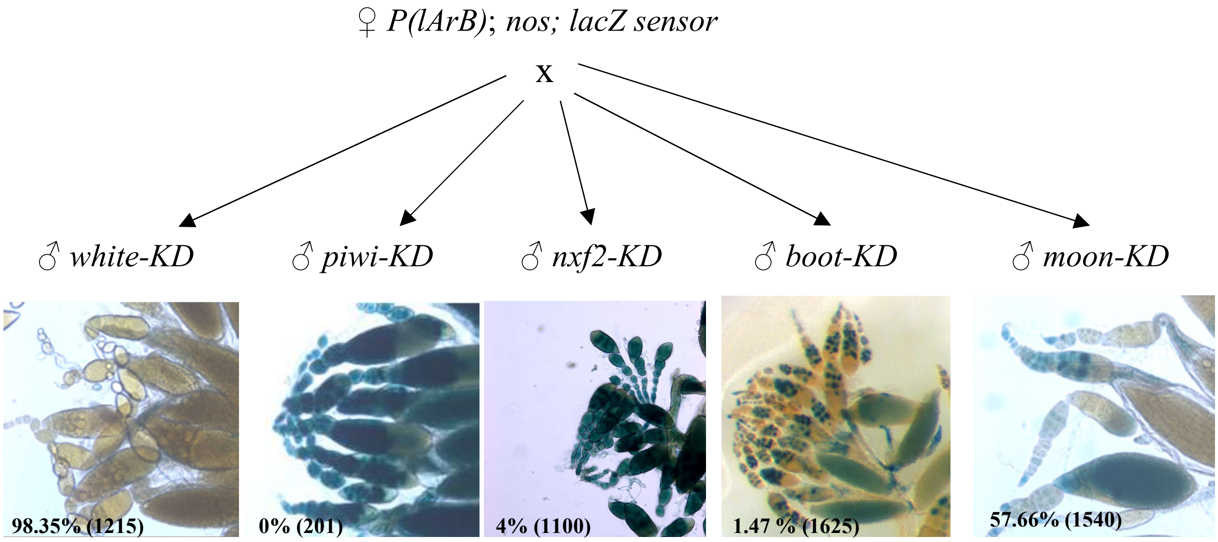

Figure S7. Ovarian *lacZ* silencing induced by *P(lArB)* is impaired by germline knockdown of genes involved in piRNA biology (*piwi*, *nxf2*, *boot, moon)*. Parental crosses are indicated above images.

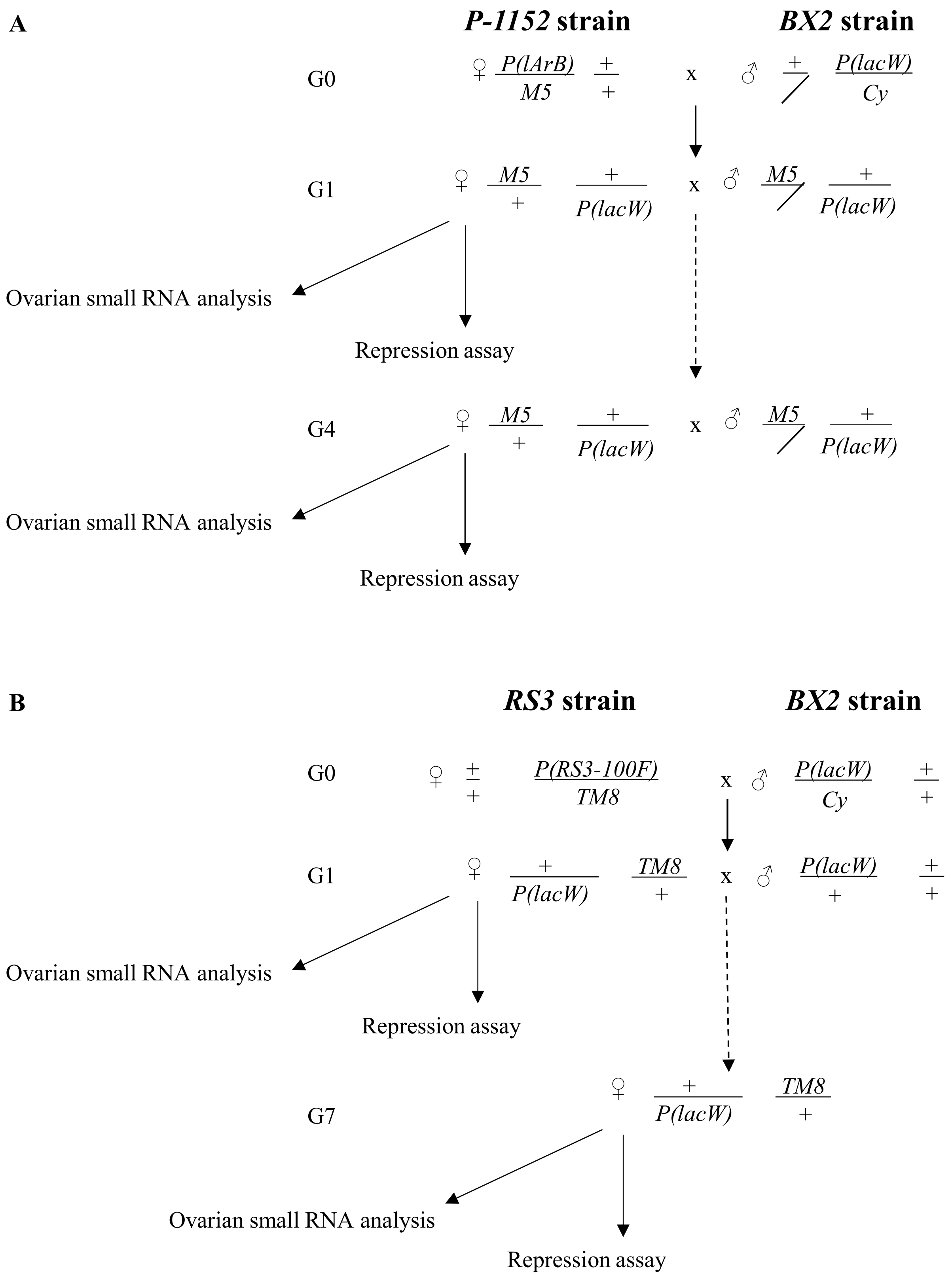

Figure S8. Crosses used for the conversion of the *P(lacW)* transgenes by *P(lArB)* or *P(RS3)*. A. Crosses between *P-1152* females, carrying maternally inherited hemizygous *P(lArB)* transgenes inserted in *cluster 1A* over the *M5* balancer chromosome, with *BX2* males carrying the heterozygous seven tandemly repeated *P(lacW)* transgenes on the *2nd* chromosome over the *Cy* balancer chromosome. **B.** Crosses between females carrying a maternally inherited *P(RS3)* transgene inserted in cluster *100F* of the 3rd chromosome over the *TM8* balancer chromosome with *BX2* males. In both crosses, G1 progenies carrying the *P(lacW)* transgenes without either the *P(lArB)* or the *P(RS3)* transgenes were recovered and crossed to each other for several generations.

Asif-Laidin et al.

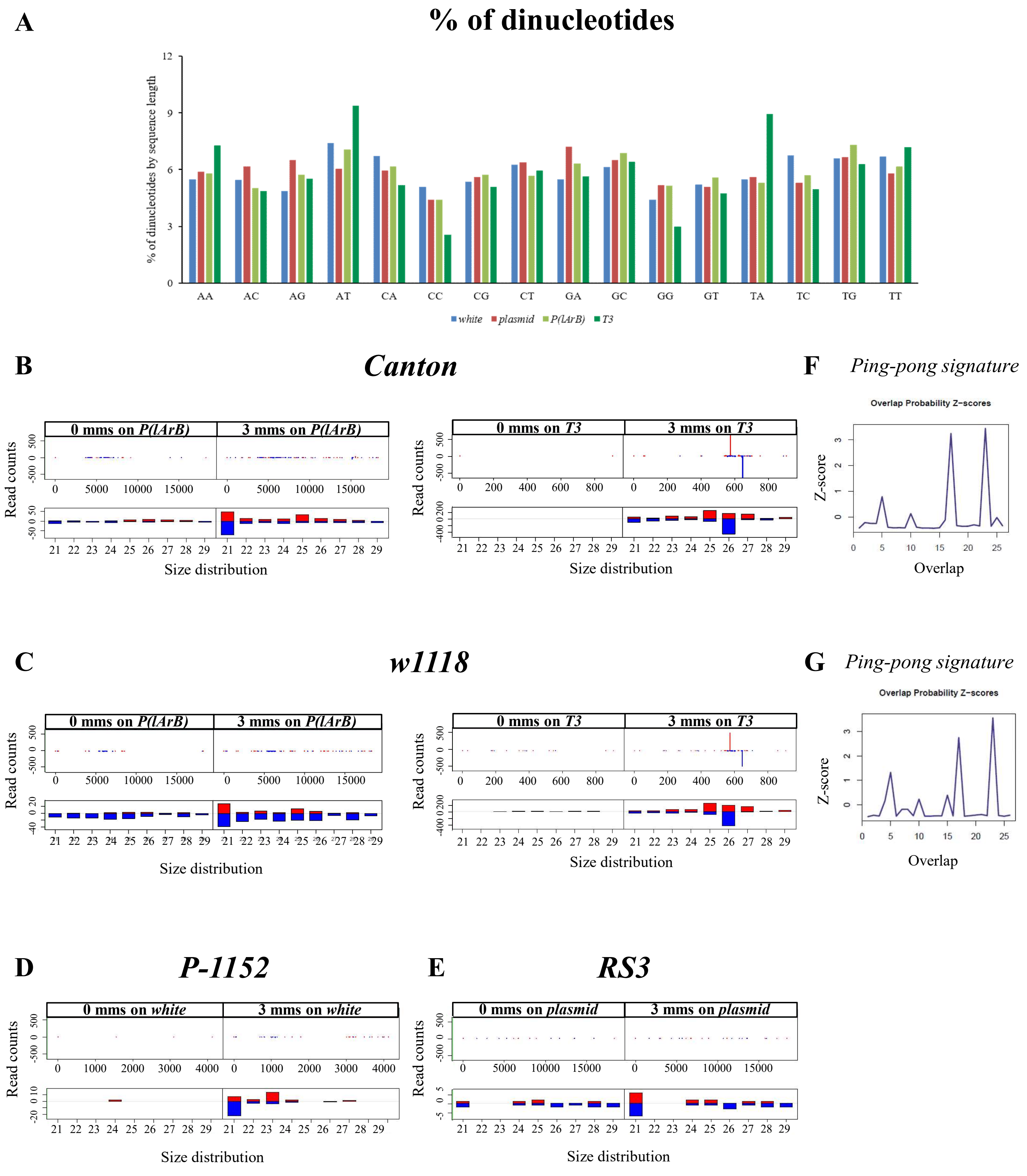

Figure S9. Study of composition of the converted sequences. A. % of dinucleotides of relevant sequences. **B-G.** Small RNAs matching the reference sequences with 3 mismatches. **B-C.** Normalized ovarian 21-29 nt RNAs mapped on *P(lArB)* and *T3* with 0 and 3 mismatches from *Canton* strain (**B**) from *w1118* strain (**C**), from *P-1152* strain mapped on *white* of the *P(lacW)* transgene with 0 and 3 mismatches (**D**), and from *RS3* strain mapped on plasmid of the *P(lacW)* transgene with 0 and 3 mismatches (**E**). **F-G.** relative frequency (z-score) of overlapping sense-antisense small RNA pairs in the subsets of 23-29 nt small RNAs with 3 mms matching *T3* in *Canton* (**F**) or in *w1118* (**G**). mms: mismatches. (see also Additional File 2: Table S6).

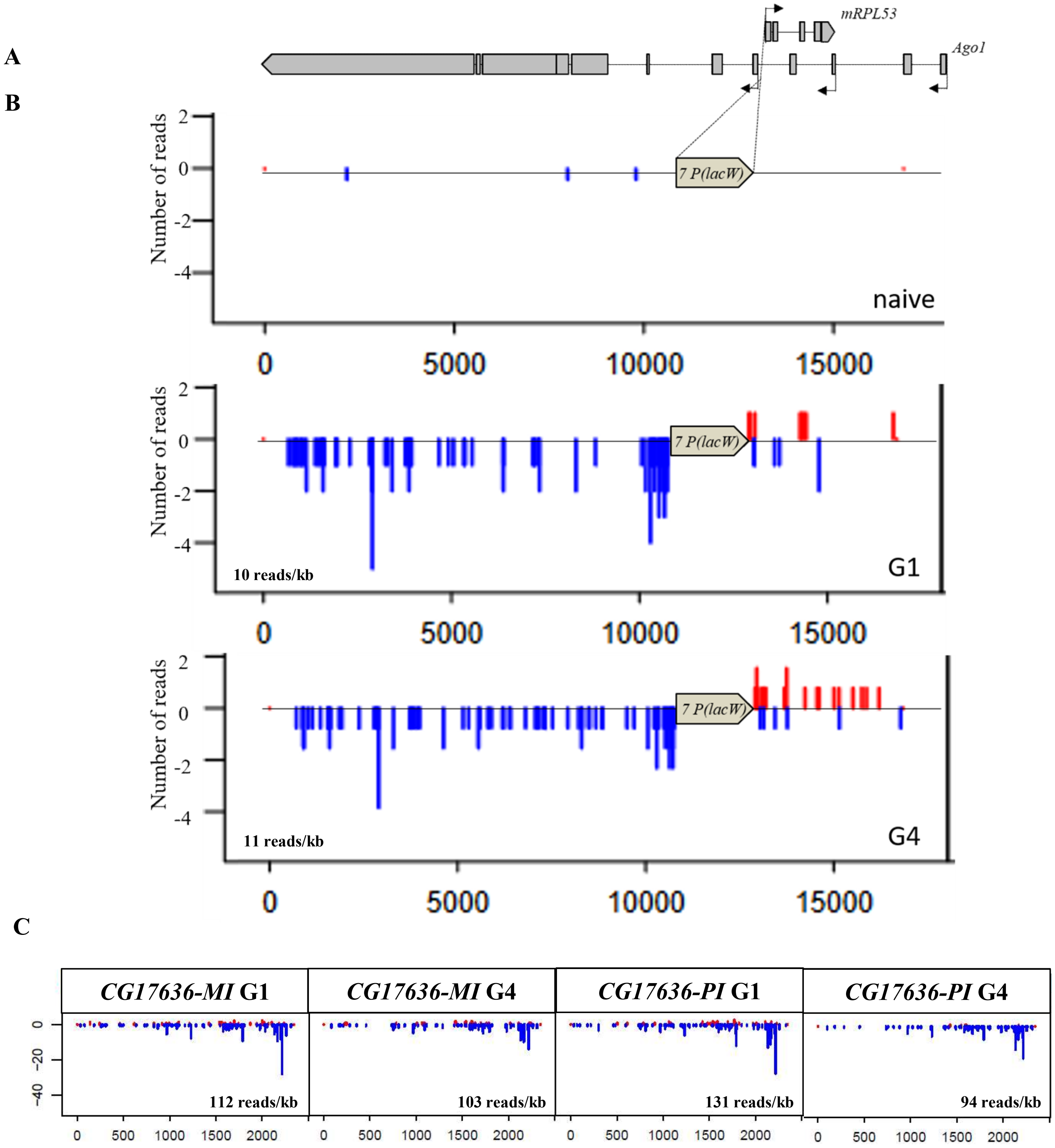

Figure S10. No *cis*-conversion of flanking regions of the *P(lacW)* transgene cluster or of *cluster 1A*. A. Schematic representation of the seven tandemly repeated *P(lacW)* transgenes inserted in tandem in the *Ago1/mRPL53* region in the *BX2* strain. Broken arrows indicate the transcription direction of *mRPL53* and *Ago1*. **B.** Normalized 23-29 nt reads matching the 5' and 3' regions of the *P(lacW)* insertion site in a naïve context or G1 and G4 after conversion by *P(lArB)* **(Fig. 4** and **S8A**). "Naïve" refers to the non-converted *P(lacW)* transgenes for piRNA synthesis. Note the scale of the Y axis as compared to the one in **Fig. 4**. **C.** Normalized 23-29 nt small RNAs were mapped to *CG17636* sequence, the most proximal gene on the *X* chromosome at G1 and G4 in *MI* and *PI* lineages. Few piRNAs are identified on flanking regions of *P(lacW)* and *P(lArB)* insertion*s*, indicating a mild impact but no conversion into a piRNA cluster of both regions. Numbers in each panel represent the density of normalized 23-29 nt reads per kb in each condition (reads/kb).

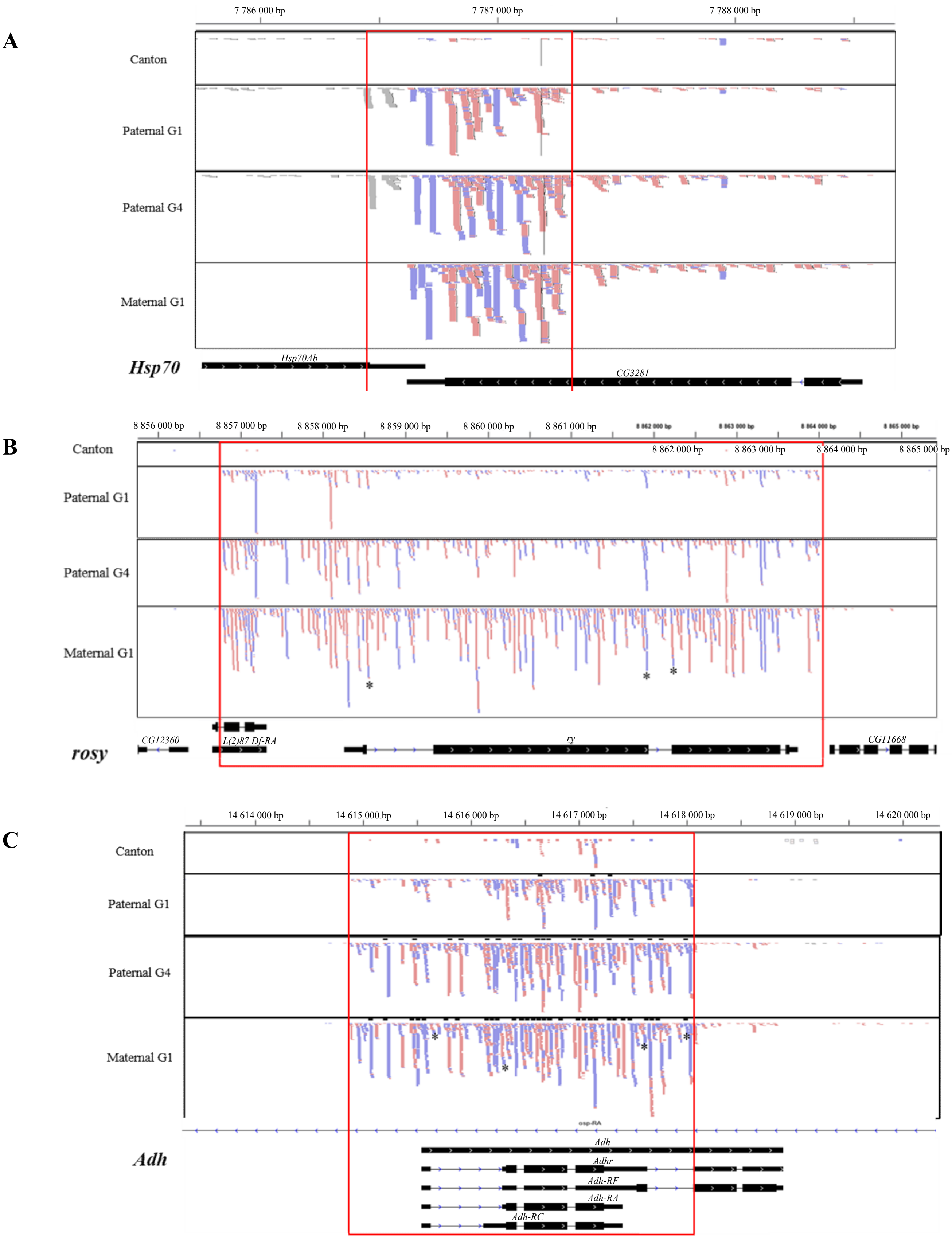

Figure S11. No *trans*-conversion of endogenous homologous sequences. A-C. The raw values of 23 to 29 nt small RNAs from *Canton*, *P(lArB)-PI* at G1 and G4 and *P(lArB)-MI* at G1 have been plotted against the *D. melanogaster* genome (R5.9) under IGV (Integrative Genomes Viewer). The red frames highlight the exact regions present on the *P(lArB)* transgene: terminal regions of *CG3281* and *Hsp70* genes (**A**), *rosy* (**B**) and *Adh* (**C**). Red and blue reads are for the sense and anti-sense reads, respectively. Grey reads correspond to reads mapping on multiple sites, here on the duplicated *Hsp70Ab* and *Hsp70Aa* genes. Black stars represent the overlapping small RNAs between exons and introns. In all three cases, the maternally inherited transgenic piRNAs have no or a minor effect on the piRNAs produced from the endogenous homologous loci.

Asif-Laidin et al.

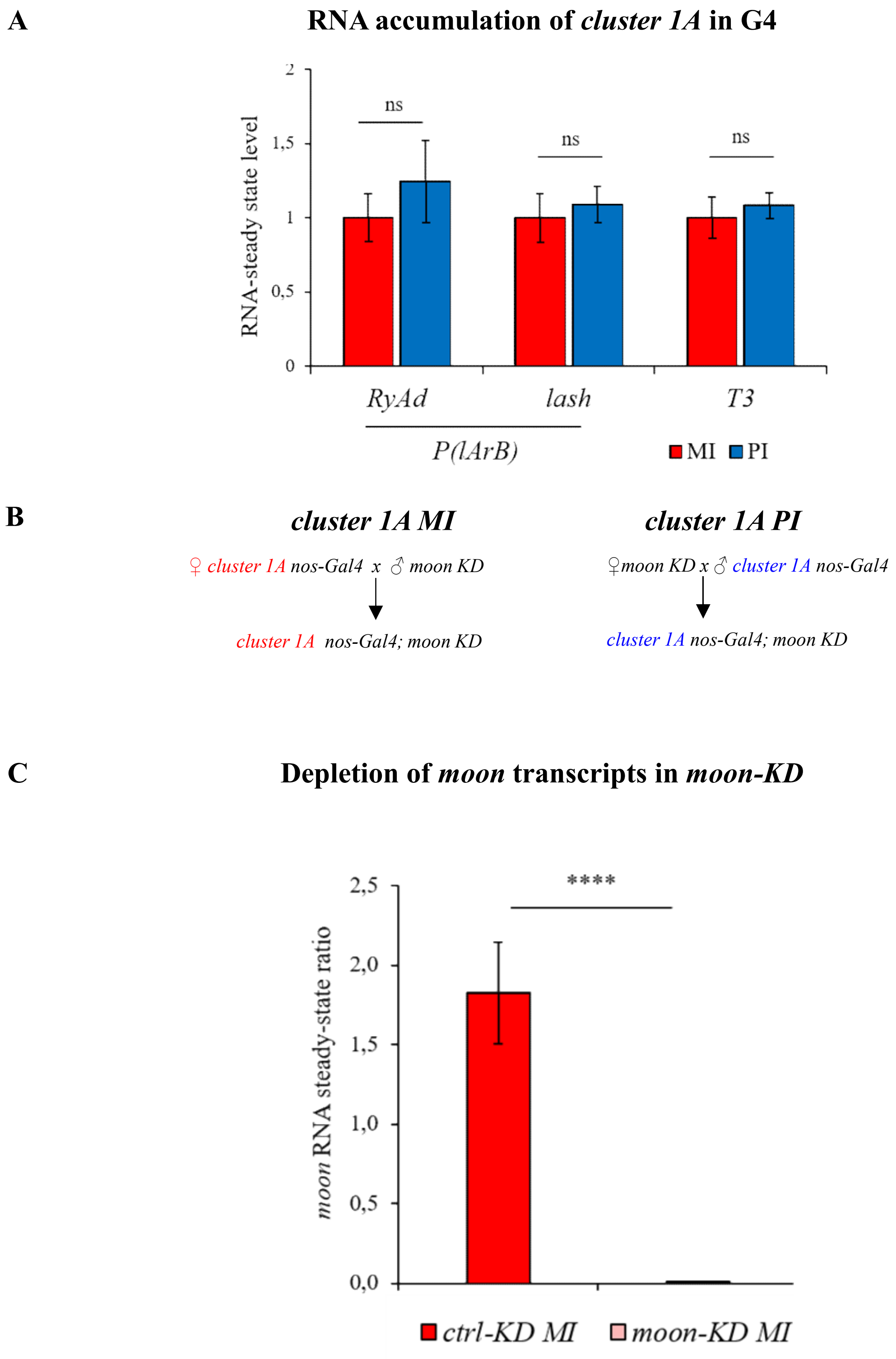

Figure S12. RT-qPCR experiments of *P(lArB)* and *T3* in *P-1152*. **A.** RT-qPCR experiments on *P(lArB)* and *T3* in *P-1152* in G4 revealed that the steady-state level of ovarian *P(lArB)* and *T3* RNAs after four generations are similar between *MI* and *PI*. RNA steady state for *MI* was normalized to 1. **B.** Crosses performed to test the effect of germline knockdown of *moonshiner* (*moon-KD*). **C.** RT qPCRs to confirm depletion of *moon* in the experimental conditions. *P*-values were calculated using a bilateral *t*-test, n=4, ns: not significant (p>0.05), **** *P*<0.0001.

A *P(lArB)-PI* D

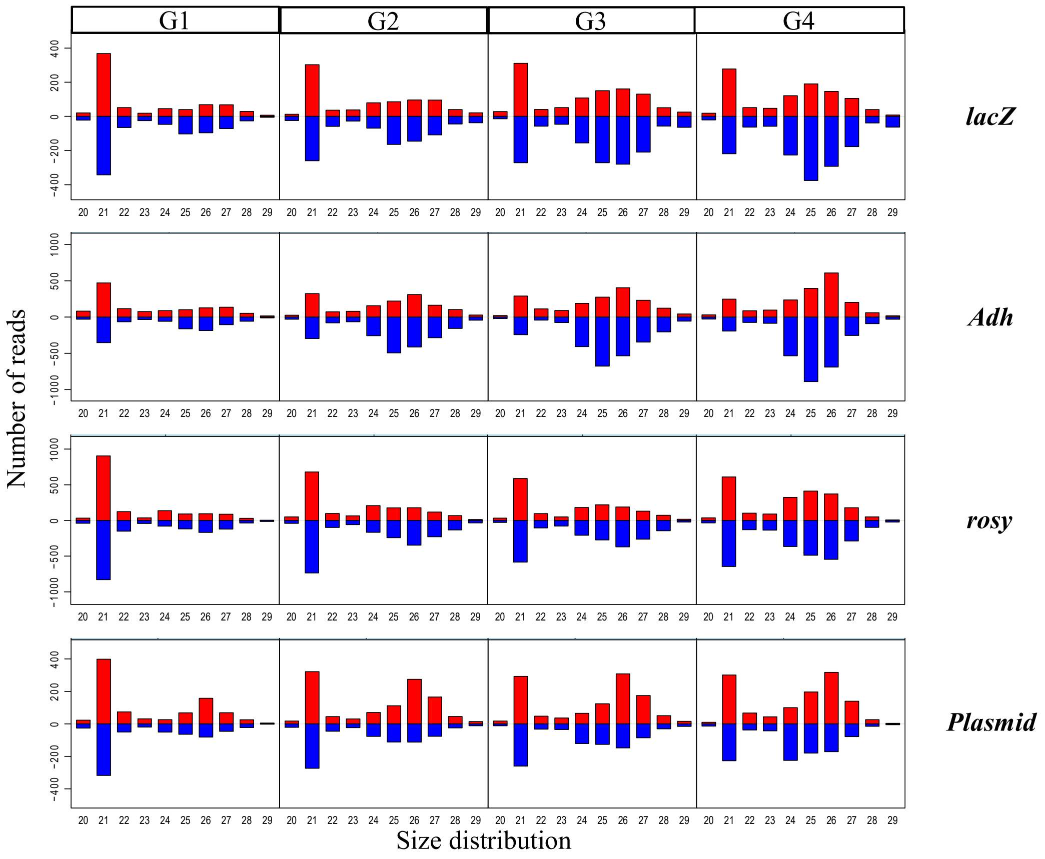

B *P(lArB)-PI* B

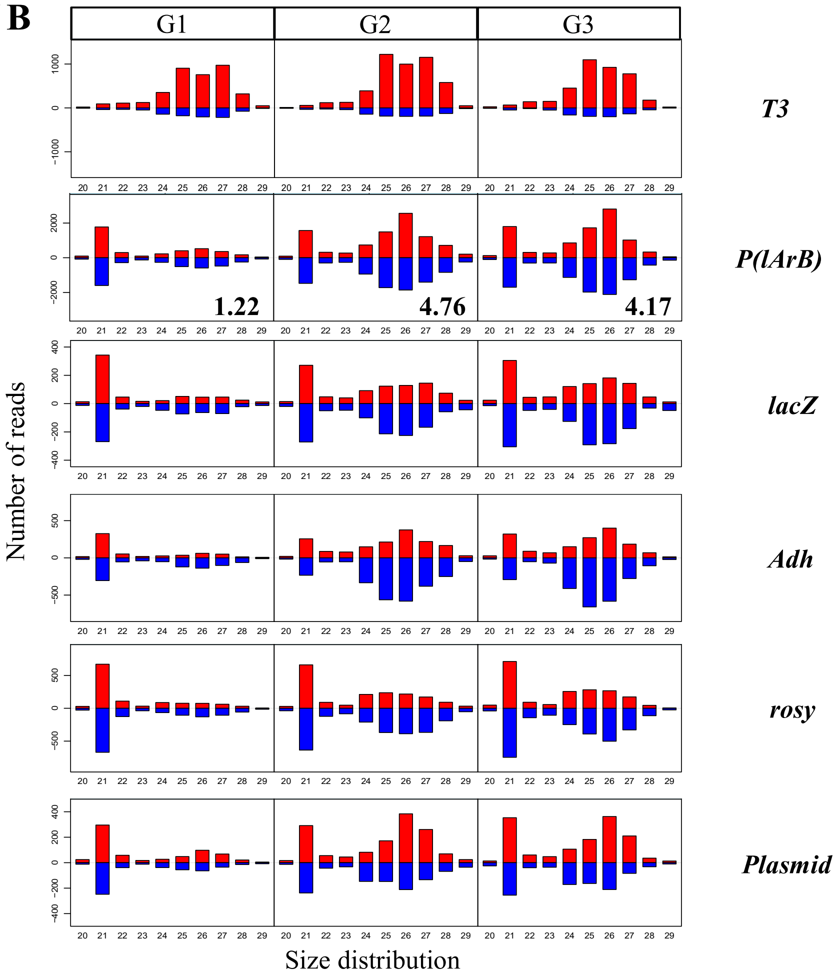

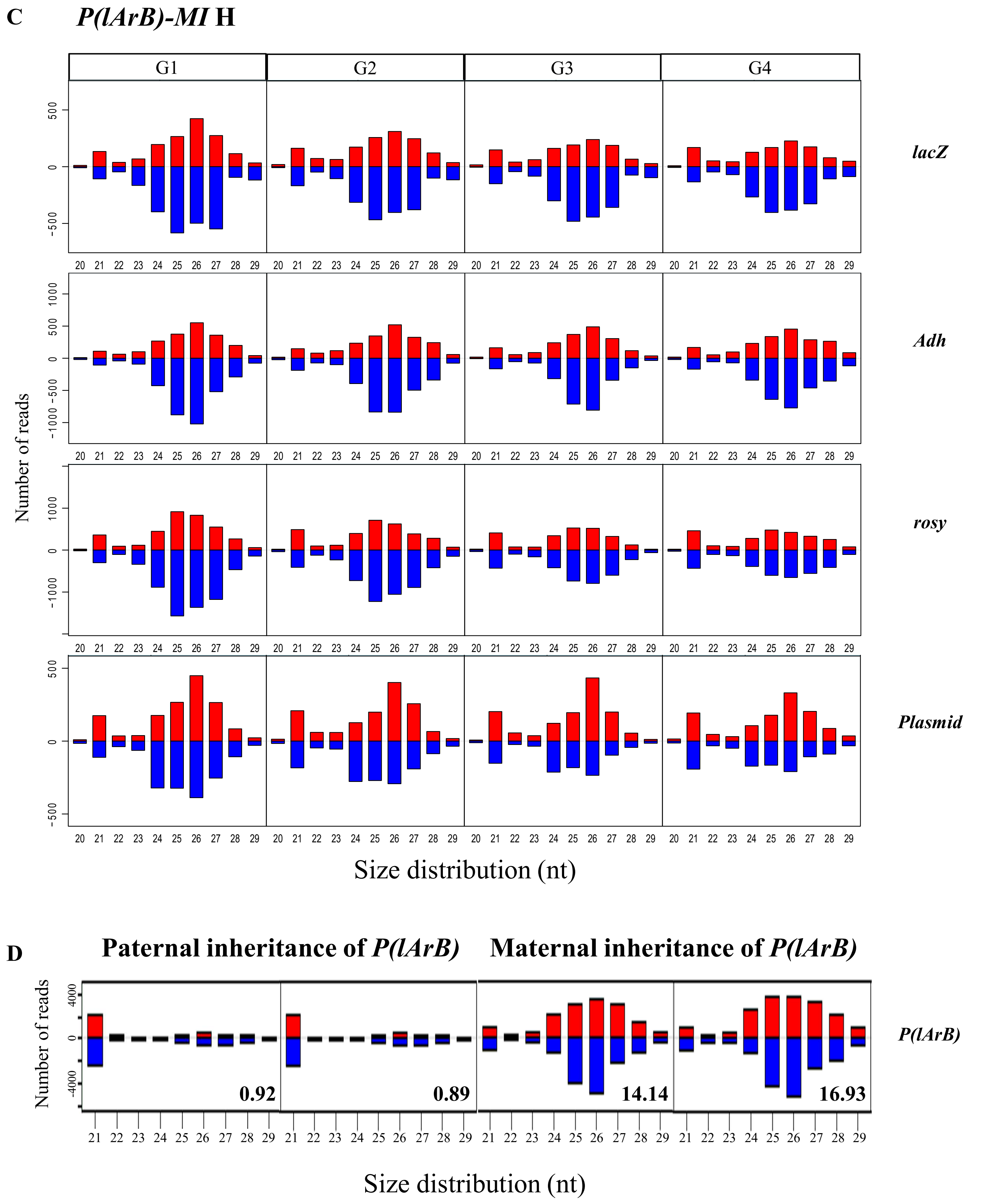

Figure S13. siRNAs and piRNAs abundance during conversion of *cluster 1A*. Size distribution of small RNAs isolated from the *P(lArB)-PI* subline D (**A**), the *P(lArB)-PI* subline B (**B**) or the *P(lArB)-MI* subline H (**C**) matching to the different domains of *P(lArB*) in *Canton* background or in *w1118* background (**D**). The numbers in panels B and D are the ratio of normalized 23-29 nt RNAs over 21 nt RNAs.

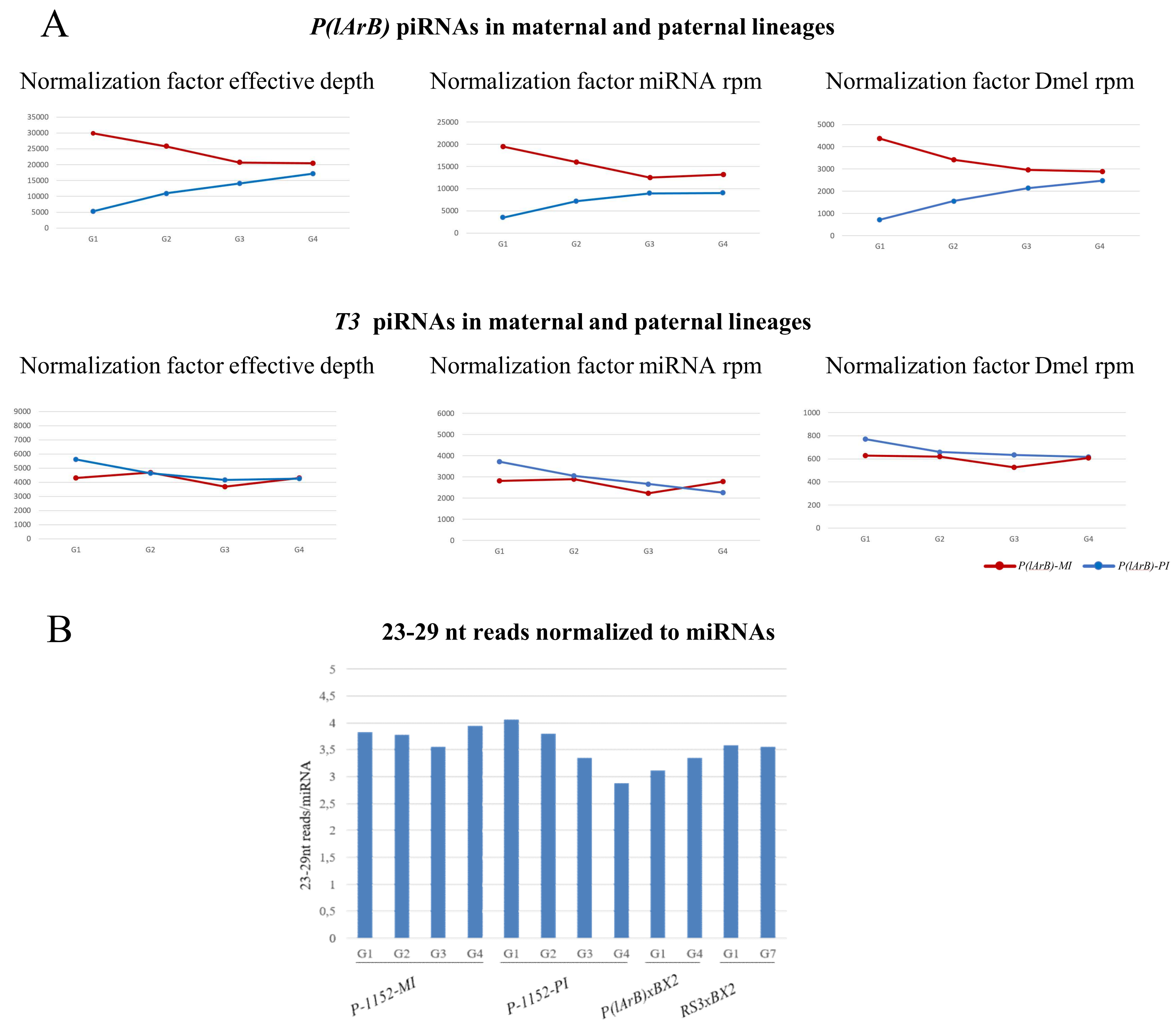

Figure S14. Comparison of methods for small RNA library normalizations. **A.** Comparison of three methods of read count normalizations performed on sublines *P-1152-MI H* and *P-1152-PI D.* **B.** 23-29 nt reads normalized to miRNAs for each libraries (Additional File 2: Table S4).
