# Supplementary material for: Modeling early germline immunization after horizontal transfer of transposable elements reveals internal piRNA cluster heterogeneity
Source: BMC Biol. 2023 May 24;21:117. doi: 10.1186/s12915-023-01616-z (PMC10210503; doi:10.1186/s12915-023-01616-z)
Supplement: Supplementary file 2 — Additional file 2: Table S1. List of Drosophila strains. Table S2. Ovarian lacZ repression for the maternally and paternally inherited Ptransgenes. Table S3. Normalized number of unique mappers of 23 to 29 reads in the 3 sublines sequenced. Table S4. Annotation of small RNA libraries. Table S5. Percentage of dinucleotides content. Table S6. Number of 21 ntand 23-29 ntreads with 0 or 3 mismatches mapping to P, T3, white and plasmid. Table S7. Primers used in this study. Table S8. P-values calculated for the H3K9me3 ovarian enrichment by ChIP-qPCR. [file 12915_2023_1616_MOESM2_ESM.pdf]

Table S1. List of *Drosophila* strains

Strains	Contains the *1A* piRNA cluster	Contains the *60F* and/or *100F* piRNA clusters	Transgenes	Localization of transgene References
P-1152	Yes	Yes	2 *P(lArB)*	piRNA *cluster 1A* [31]
Canton	No	Yes	No transgene	-
w^{1118}	No	Yes	No transgene	-
Oregon	Yes	Yes	No transgene	-
BX2	No	Yes	7 tandemly repeated *P(lacW)*	Euchromatic (*50C*) [12]
RS3	No	Yes	*P(RS3)*	piRNA *cluster 100F* [62]
nosGal4	No	NT	PBac{w[+mW.hs]=GreenEye.nosGAL4}	Third chromosome [32]
pRFP	No	NT	*pUASp-RFP*	Third chromosome (this study)
pRFP-T3	No	NT	*pUASp-RFP* transcriptionally fused to *T3* sequence; *T3* piRNA sensor	Third chromosome (this study)
lacZ sensor	No	NT	*BQ16: P(92)* encoding an euchromatic *lacZ* piRNA sensor	Third chromosome (*64C*) [31]
P(lArB); nos; lacZ sensor	Yes	NT	2 *P(lArB), nosGal4, BQ16*	piRNA *cluster 1A* and third chromosome (this study)
Oregon nos; pRFP-T3	Yes	NT	*nosGal4, BQ16, pRFP-T3*	Third chromosome (this study)
piwi RNAi	No	NT	*VDRC 22235*	Third chromosome FBst0454485
moon sh	No	NT	*PA61*	Third chromosome [10]
white sh	No	NT	*BDSC 33644*	Third chromosome [65]
nxf2 sh	Non	NT	*BDSC 34957*	Third chromosome [45]
boot sh	Non	NT	*BDSC 36610*	Third chromosome [45]

NT: Not Tested. These transgenic lines were generated in a w^{1118} genetic background. It is therefore assumed that they possess only autosomal subtelomeric piRNA clusters.

Table S2. Ovarian *lacZ* repression for the maternally and paternally inherited *P(lArB)* transgenes

	Paternally transmitted *P(lArB)* silencer transgenes: *P(lArB)-PI*				Maternally transmitted *P(lArB)* silencer transgene: *P(lArB)-MI*			
Sublines	A	B	C	D	E	F	G	H
G1	9.25% (n=1000)				93.5% (n=1000)			
G2	55.7% (n=1100)	44.35% (n=1100)	42.5% (n=1350)	41.2% (n=750)	89.7% (n=1100)	85.1% (n=1350)	86.75% (n=1450)	87.6% (n=1550)
G3	82.25% (n=1300)	83.75% (n=1500)	75.1% (n=1400)	76.25% (n=1450)	96.75% (n=1350)	93.55% (n=1400)	91.8% (n=1500)	98.0% (n=1400)
G4	96.3% (n=1400)	90.5% (n=1350)	91.95% (n=1300)	95.1% (n=1600)	91.35% (n=1800)	88.1% (n=1650)	84.15% (n=1700)	86.15% (n=1350)
G5	95.93% (n=1350)	NT	89.6% (n=600)	88.35% (n=1300)	92.4% (n=1550)	85.55% (n=1250)	NT	90.9% (n=1250)
G6	94.5% (n=850)	92.1% (n=600)	92.7% (n=1050)	98.0% (n=1000)	91.8% (n=850)	91.18% (n=800)	96.9% (n=1000)	91.85% (n=650)
G7	77.4% (n=1200)	98.75% (n=1200)	NA	NA	89.5% (n=1100)	80.0% (n=2000)	NA	71.0% (n=1550)
G8	13.5% (n=1450)	73.5% (n=1000)	NA	NA	78.1% (n=1350)	69.75% (n=800)	NA	73.1% (n=1450)
G9	46.4% (n=700)	87.4% (n=700)	NA	NA	96.5% (n=800)	79.5% (n=400)	NA	

To measure the levels of *lac Z* repression, females of each cross shown in **Additional file 1: Fig. S2** were crossed with males carrying a euchromatic *P-lacZ* sensor transgene expressed in female germline. *LacZ* germline repression was scored by calculating the % of repressed egg chambers. Twelve females were tested for each cross. n: number of total egg chambers counted.

NT: Not Tested

NA: Not Applicable, when the subline was sterile

Table S3. Normalized number of unique mappers of 23 to 29 reads in the 3 sublines sequenced.

P(lArB)-MI H	*P(lArB)*	*T3* domain	*lacZ*	*Hsp70*	*Adh*	*rosy*	*Plasmid*	*P5′*	*P3′*	*P5′+P3′*
Size (kb)	18.292	0.9	3.047	0.9	3.155	7.071	2.688	0.586	0.228	0.814
G1	30211.74	4252.56	3774.42	7861.62	5204.16	9133.80	2786.94	698.88	343.2	1042.08
G2	26079.36	4624.80	3091.66	6969.16	4926.54	7284.06	2334.96	717.22	383.52	1100.74
G3	20991.64	3658.84	2771.04	6275.32	4085.72	4928.44	1874.04	593.4	276	869.4
G4	20680.00	4275.00	2512.00	5900.00	4516.00	4782.00	1796.00	556	302	858

P(lArB)-PI D	*P(lArB)*	*T3* domain	*lacZ*	*Hsp70*	*Adh*	*rosy*	*Plasmid*	*P5′*	*P3′*	*P5′+P3′*
G1	5394	5012	649.02	1233.66	1208.43	1057.92	667.29	254.91	247.08	501.99
G2	11035.64	4562.25	1051.05	3391.85	2769.69	2769.69	1148.84	301.84	219.45	521.29
G3	14109.17	4098.54	1733.87	4447.14	3605.52	3605.52	1319.7	415	238.21	653.21
G4	17277.57	4223.94	1888.58	5498.42	4183.89	4183.89	1543.26	359.56	244.75	604.31

P(lArB)-PI B	*P(lArB)*	*T3* domain	*lacZ*	*Hsp70*	*Adh*	*rosy*	*Plasmid*	*P5′*	*P3′*	*P5′+P3′*
G1	4117.36	4360.08	526.14	990.86	738.52	882.08	510.60	219.78	202.02	421.8
G2	14475.14	5405.70	1481.48	3955.30	3445.44	2666.22	1812.26	522.44	389.24	911.68
G3	14433.65	4376.68	1691.69	4082.54	3284.05	2795.10	1663.20	366.52	314.16	680,68

**Control Strains**	*P(lArB)*	*T3* domain	*lacZ*	*Hsp70*	*Adh*	*rosy*	*Plasmid*	*P5′*	*P3′*
Canton (G0)	60	0	0	24	31	4	1	0	0
P-1152 (G0)	40769	7477	4773	8090	7825	13807	4316	618.4	502.4

Normalization factors are found in Additional Files 2: **Table S4**.

Table S4: Annotation of small RNA libraries

Small RNAs were prepared from ovaries of females of the indicated strains. Values for the different categories of sequences matching to the *D. melanogaster* genome 5.49 in each library are indicated. For comparisons, libraries were normalized (normalization factor) relatively to the number of sequence reads aligning to the *D. melanogaster* genome but not to miscRNAs (including rRNA and snoRNA) or tRNAs (effective depth) (Asif-Laidin et al., 2017). The libraries with the lowest effective depth were taken as reference to normalize the other libraries (**A**. library #4, **B**. library #1, **C**. library #1, **D**. library #1, **E**. library #1). Libraries were also normalized relatively to the number of sequence reads aligning to miRNAs or to the *D. melanogaster* genome (rpm) for comparison of the three methods.

**A. Libraries**	1	2	3	4	5	6	7	8	9	10	11	12	13
**Alignment reference**	GRH117	GRH118	GRH119	GRH120	GRH121	GRH122	GRH123	GRH124	GRH126	GRH127	GRH128	GRH129	GRH133
**Subline**	**Maternal**				**Paternal**								
**Generations**	G1	G2	G3	G4	B G1	D G1	B G2	D G2	B G3	D G3	D G4	*Canton*^{y}	*P-1152*
***D. melanogaster* (Flybase r5.49)**	8749771	8010061	7614649	7092541	8792420	8365118	9448234	9092202	9421670	7925059	7751589	10153341	10011295
**Dmel_all-miscRNA**	401121	964415	502779	542585	5455	839017	550691	764979	908722	55603	454261	924278	1416324
**Dmel_all-tRNA**	122722	215323	108824	118454	63501	138242	192744	20465	212698	117016	99062	107897	557997
**Dmel_all-transposon**	4206089	3362562	3447748	3219305	4058452	3707867	4502347	4084503	4100466	3537130	3417567	4423806	4067114
**Dmel_miR_r20**	1959665	1714231	1803742	1546512	1998328	1732825	1879526	1965293	2049340	1885826	2119232	1638666	1235457
**23-29-nt reads**	7511438	6481051	6424246	6094464	7361451	7027368	8176776	7473551	7547860	6328200	6106725	8283029	8346991
**Remaining Unmatched**	2060174	1753530	1751556	1665685	2126639	1947167	2322926	2072777	2150444	1829057	1661467	477717	477418
**Effective depth**	8225928	6830323	7003046	6431502	8723464	7387859	8704799	8306758	8300250	7752440	7198266	9121166	8036974
**Normalization factors**													
**on effective depth**	0.78	0.94	0.92	1.00	0.74	0.87	0.74	0.77	0.77	0.83	0.89	0.71	0.80
**On one Mio miRNAs (rpm)**	0.510	0.583	0.554	0.647	0.500	0.577	0.532	0.509	0.488	0.530	0.472	0.610	0.809
**On one Mio Dmel (rpm)**	0.114	0.125	0.131	0.141	0.114	0.120	0.106	0.110	0.106	0.126	0.129	0.098	0.100
**23-29-nt reads /miRNAs**	3.83	3.78	3.56	3.94	3.68	4.06	4.35	3.80	3.68	3.36	2.88		

**B. Libraries**	1	2	3	4
**Alignment reference**	GRH97	GRH98	GRH99	GRH100
**Subline**	P1152 x W1118	P1152 x W1118	W1118 x P1152	W1118 x P1152
***D. melanogaster* (Flybase r5.49)**	8972279	10144872	12760054	12631941
**Dmel_all-miscRNA r5.49**	1159528	1422030	990966	1152945
**Dmel_all-tRNA r5.49**	81301	101940	101888	93653
**Dmel_all-transposon-r5.49**	4344546	4972906	6732299	6620332
**Dmel_miR_r20**	2069196	2130922	2986509	2855154
**Effective depth**	7731450	8620902	11667200	11385343
**Normalization factor**	1.00	0.90	0.66	0.68

**C. Libraries**	1	2
**Alignment reference**	GRH15	GRH16
**Subline**	RS3 x BQ16	BQ16 x RS3
***D. melanogaster* (Flybase r5.49)**	19991599	22763040
**Dmel_all-miscRNA r5.49**	3380686	2941295
**Dmel_all-tRNA r5.49**	1102954	1502668
**Dmel_all-transposon-r5.49**	6457261	7217350
**Dmel_miR_r20**	4382403	5669436
**Effective depth**	15507959	18319077
**Normalization factor**	1.00	0.85

**D. Libraries**	1	2	3
**Alignment reference**	GRH107	GRH108	GRH12
**Subline**	BX2_P1152	BX2_P1152	BX2/ +
**Generations**	G1	G4	naïve
***D. melanogaster* (Flybase r5.49)**	7862750	10357598	20140166
**Dmel_all-miscRNA r5.49**	526863	806014	1393137
**Dmel_all-tRNA r5.49**	160045	250354	1101672
**Dmel_all-transposon-r5.49**	3938988	5213229	7687715
**Dmel_miR_r20**	1931216	2370154	6848463
**23-29-nt counts**	6010941	7938722	
**Effective depth**	7175842	9301230	17645357
**Normalization factor**	1.00	0.77	0.41
**23-29-nt reads /miRNAs**	3.11	3.35	

**E. Libraries**	1	2
**Alignment reference**	GRH150	GRH148
**Subline**	RS3_BX2	RS3_BX2
**Generations**	G1	G7
***D. melanogaster* (Flybase r5.49)**	10462432	10975285
**Dmel_all-miscRNA r5.49**	1134092	1298089
**Dmel_all-tRNA r5.49**	556522	538921
**Dmel_all-transposon-r5.49**	4627974	4849380
**Dmel_miR_r20**	2228288	2353898
**23-29-nt counts**	7980729	8357928
**Effective depth**	8771818	9138275
**Normalization factor**	1.00	0.96
**23-29-nt reads /miRNAs**	3.58	3.55

**F. Library**	*w1118*
**Alignment reference**	GRH93
***D. melanogaster* (Flybase dm6)**	7910948
**Dmel_all-transposon-BDGPv941**	2919004
**23-29-nt counts**	5420368

Table S5. Percentage of dinucleotides content

	white	Plasmid	*P(lArB)-PI* D	*T3*
AA	5.5004	5.8952	5.7894	7.2848
AC	5.4519	6.1681	5.0186	4.8565
AG	4.8704	6.4956	5.7402	5.5188
AT	7.3904	6.0590	7.0523	9.3819
CA	6.7119	5.9498	6.1830	5.1876
CC	5.0884	4.4214	4.4118	2.5386
CG	5.3550	5.6223	5.7293	5.0773
CT	6.2757	6.3865	5.6855	5.9603
GA	5.5004	7.2052	6.3252	5.6291
GC	6.1304	6.4956	6.8773	6.4018
GG	4.4100	5.1856	5.1443	2.9801
GT	5.2096	5.0764	5.5817	4.7461
TA	5.5004	5.6223	5.3029	8.9404
TC	6.7604	5.2948	5.6965	4.9669
TG	6.6150	6.6594	7.3201	6.2914
TT	6.6877	5.7860	6.1830	7.1744

Table S6. Number of 21 nt (siRNAs) and 23-29-nt (piRNAs) reads with 0 or 3 mismatches mapping to *P(lArB)*, *T3*, *white* and plasmid

Parental G0 strains	*Canton*		*w1118*		*P-1152*	*RS3*
Targeted sequence	*P(lArB)* 18 kb	*T3* 0.9 kb	*P(lArB)*	*T3*	*white P(lacW)* 4.1 kb	plasmid *P(lacW)*) 1.8 kb
piRNAs with 0 mms	60 (3.3)	0	96 (5.3)	29 (32.2)	2 (0.5)	13 (7.2)
piRNAs with 3 mms	147 (8.1)	1407 (1563.3) No ping-pong	172 (9.5)	1241 (1378.8) No ping-pong	24 (5.8)	16 (8.8)
siRNAs with 0mms	14 (0.8)	0 (0)	11 (0.6)	0 (0)	0 (0)	3 (1.6)
siRNA with 3 mms	119 (6.6)	159 (176.6)	68 (3.7)	67 (74.4)	29 (7.1)	13 (7.2)
sensor silencing	No *P-lacZ* silencing	NT[a]	No *P-lacZ* silencing	No *pRFP-T3* silencing		

mms: mismatches

In bracket are the number of piRNAs per kb

NT[a]: Not Tested, for technical issues

Size distribution and mapping are found in the Additional file 1: **Fig. S9B-E**.

Table S7. Primers used in this study

Primers	Sequences 5′ to 3′
RyAd For	GGGATCTGCGCCACATCG
RyAd Rev	CGGGCTGCAGGAATTCGATA
Lash For	CTTTGGCCTTAGTCGACGGA
Lash Rev	TCCTGGAGCCCGTCAGTATC
lacZ2 For	ACTATCCCGACCGCCTTACT
lacZ2 Rev	GTGGGCCATAATTCAATTCG
42AB For	AAGACCCAATTTTTGCGTCGC
42AB Rev	CAAGGATAGGGATTTGGTCC
RpL32 For	CCGCTTCAAGGGACAGTATCTG
RpL32 Rev	ATCTCGCCGCAGTAAACGC
Moon For	TCGATCATCACATCCTCCGG
Moon Rev	AGAACCAGTCATTTCAACCACA
TAS_X3_1 For	CGTATGCGAGAGGAGTGTCA
TAS_X3_1 Rev	TCCAACGCCCTAAAGAGAGA

Table S8. *P*-values calculated for the H3K9me3 ovarian enrichment by ChIP-qPCR (Fig. 5B)

Tested regions	Measure *P*-value	Symbol used on Fig. 5B
RyAd	0.02	*
lash	0.0006	***
lacZ	0.01	**
T3	0.004	**
RpL32	0.869	ns
